# In Situ Insights into Ni Phyllosilicate Evolution: Cationic Ni Species as Key to Enhanced Stability in Methane-Rich Dry Reforming

**DOI:** 10.1021/acscatal.5c07192

**Published:** 2026-02-09

**Authors:** Katarzyna Świrk Da Costa, Paulina Summa, Marco Fabbiani, Dumitrita Spinu, Valentin Valtchev, Ludovic Pinard, Magnus Ro̷nning

**Affiliations:** † 8018 Norwegian University of Science and Technology, Department of Chemical Engineering, Trondheim 7491, Norway; ‡ Sorbonne Université, CNRS UMR 7190, Institut Jean Le Rond d’Alembert, Saint Cyr l’Ecole 78210 , France; § Université de Caen, ENSICAEN, CNRS UMR 6506, Laboratoire Catalyse et Spectrochimie, Caen 14000 , France

**Keywords:** catalysts, dry reforming, nickel phyllosilicate, nanoparticles, mesoporous silica

## Abstract

Nickel (Ni) phyllosilicate-derived catalysts have recently gained attention for the CO_2_ reforming of methane. However, understanding of the underlying reduction pathways and structural factors that determine stable catalytic performance is still missing. Herein, we developed a one-pot synthesis with ammonia solution to produce nickel catalysts supported on silica, utilizing a modified KIT-6 protocol. Under the proposed alkaline conditions (pH = 9), the silanol groups were deprotonated (Si–O^–^) and the resulting negatively charged oxide surface could interact with Ni^2+^. This approach facilitated the in situ formation of Ni phyllosilicate within the silica framework, which contained isolated surface hydroxyl groups. In situ XAS-XRD revealed the presence of thermally stable crystalline Ni phyllosilicate, Ni_3_Si_2_O_5_(OH)_4_, with time-resolved XANES providing complementary insight into the redox transformation of nickel species associated with dehydroxylation. Partially unreduced nickel species retained a cationic state during the catalytic reaction at 700 °C, with a higher amount of nickel phyllosilicate observed after 50 h in contrast to the state after 1 h. On the whole, the one-pot synthesis produced small Ni crystallites with improved dispersion, both of which had a part in ensuring stable catalytic performance. We also uncovered the crucial role of ionic species (Ni^+^ and Ni^2+^) limiting the carbon formation via CO disproportionation (2CO ⇌ C_(s)_ + CO_2_) on the KIT-6-templated silica. This study provides valuable insights into the design of more stable methane reforming catalysts.

## Graphical abstract

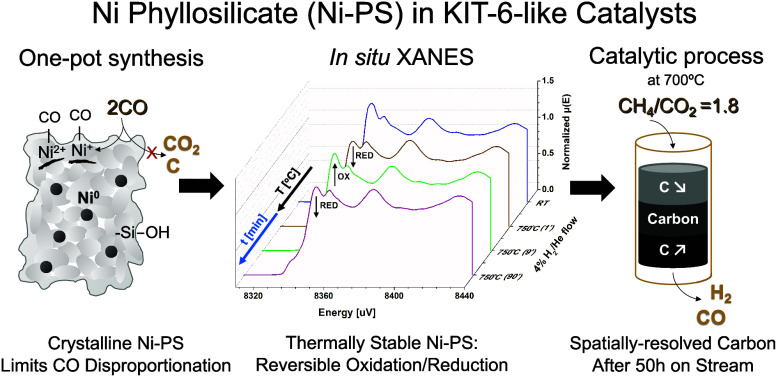

## 1 Introduction

Dry (CO_2_) reforming of methane (DRM) (eq 1) has emerged as the most widely studied reaction to turn CH_4_ and CO_2_ into synthesis gas, known as a versatile feedstock for chemical synthesis and synthetic fuels.

$${\mathrm{CH}}_{4}+{\mathrm{CO}}_{2}⇌2H_{2}+2\mathrm{CO}\;\;\;\Delta H_{298\;K}^{0}=247\;\mathrm{kJ}{\mathrm{mol}}^{‐1}$$ 1

An alternative to the conventional dry reforming of methane (DRM) is methane-rich dry reforming, offering a pathway for upgrading low-quality natural gas (a mixture of CH_4_ containing significant amounts of CO_2_). This may be the case of some natural gas wells in Hungary (e.g., Méhkerék Gas Field),^1^ Austria (e.g., Vienna Basin),^2^ or Norway (e.g., Sleipner Field).^3^

Under both stoichiometric and nonstoichiometric feed conditions, the dry reforming of methane is accompanied by several parallel reactions that promote carbon formation, such as

methane decomposition:

$$\begin{array}{cc} {\mathrm{CH}}_{4}⇌C_{\left(s\right)}+2H_{2} & \Delta H_{298\;K}^{0} \end{array}=75\;\mathrm{kJ}{\mathrm{mol}}^{‐1}$$ 2

Boudouard reaction (CO disproportionation):

$$\begin{array}{cc} 2\mathrm{CO}⇌C_{\left(s\right)}+{\mathrm{CO}}_{2} & \Delta H_{298\;K}^{0}=-172\;\mathrm{kJ}{\mathrm{mol}}^{‐1} \end{array}$$ 3

CO_2_ hydrogenation:

$$\begin{array}{cc} {\mathrm{CO}}_{2}+2H_{2}⇌C_{\left(s\right)}+2H_{2}O & \Delta H_{298\;K}^{0}=-90\;\mathrm{kJ}{\mathrm{mol}}^{‐1} \end{array}$$ 4

CO hydrogenation:

$$\begin{array}{cc} \mathrm{CO}+H_{2}⇌C_{\left(s\right)}+H_{2}O & \Delta H_{298\;K}^{0}=-131\;\mathrm{kJ}{\mathrm{mol}}^{‐1} \end{array}$$ 5

Carbon deposited through these pathways may be oxidized or gasified via the reverse reactions—namely carbon hydrogenation (inverse eq 2), the reverse Boudouard reaction (inverse eq 3), and steam gasification (inverse eqs 4 and 5)—thereby reducing net coking and helping maintain catalytic activity.

Moreover, other side reactions, such as the reverse water–gas shift (eq 6), may also occur, modifying the H_2_/CO molar ratio and resulting in higher CO_2_ conversion:

Reverse water–gas shift (RWGS):

$$\begin{array}{cc} {\mathrm{CO}}_{2}+H_{2}⇌\mathrm{CO}+H_{2}O & \Delta H_{298\;K}^{0}=41\;\mathrm{kJ}{\mathrm{mol}}^{‐1} \end{array}$$ 6

Nickel, as one of the most often used transition metals in DRM, not only delivers high catalytic performance but it is also abundant and more cost-effective than noble metals, making the reforming process more economically viable.^4,5^ More recent in situ studies demonstrated that surface carbon formed by methane decomposition (eq 2) on Ni catalysts can act as active reaction intermediates via reverse Boudouard reactions (inverse eq 3) for syngas production, instead of deactivating the catalyst.^6,7^ This claim is supported by Wei and Iglesia, who showed that CH_4_ and CO_2_ are activated in subsequent steps.^8,9^ Nevertheless, if the rate of carbon formation exceeds those of C removal, then deactivation can occur through encapsulating carbon layers or filament growth. In addition to carbon deposition, Ni catalysts may also suffer from sintering, particle agglomeration, or oxidation of the metallic phase under DRM conditions,^10^ emphasizing the need for strategies to stabilize both the metal Ni nanoparticles and the carbon oxidation pathways.

Silica-supported nickel systems have been widely explored in reforming of methane reactions due to high thermal stability at the temperatures of 700–900 °C. Although Ni-SiO_2_ is one of the most prevalent choices, it usually suffers from weak interactions between nickel species and the silica support, in consequence leading to Ni agglomeration and/or sintering at high temperatures and therefore inferior stability.^4^

Several efficient mesoporous silicas (SBA-15, MCM-41, and KIT-6) were proposed to overcome the aforementioned problem. Among them, KIT-6 gained recognition, owing to the distinctive structural properties within the 3D continuous cubic structure with interconnected mesopores.^11–13^ KIT-6 has a remarkably high surface area of ca. 750 m^2^/g, enabling nickel particles to obtain improved dispersion and uniform surface distribution. Ni-KIT-6 silica catalysts are commonly prepared through postsynthesis impregnation of the silica support with an aqueous nickel solution; hence, such materials were previously studied by us as well. Dry impregnation^14^ and wet impregnation^15^ methods were applied, and the performance of the resulting catalysts in dry reforming of methane (CH_4_/CO_2_/Ar) was valorized. Both impregnation methods resulted in the formation of relatively large nickel particles, which are prone to carbon formation under the reaction conditions. As a step toward improvement, a one-pot synthesis is proposed. In principle, the method allows strengthening the metal–support interactions (MSIs) and preserving the smaller size of metallic particles of Ni.^16–18^ The remaining challenge linked with this method lies in the pH adjustment to promote coprecipitation of the Ni precursor and the simultaneous formation of the mesoporous 3D structure typical of KIT-6. Nickel­(II) nitrate hexahydrate typically precipitates when pH of the solution is raised to ca. 8–9, while acidic environment (pH 1.5–2) is necessary for proper formation of the cubic mesoporous *Ia3 ®d* structure of KIT-6.^19^ Under strongly acidic conditions, the silanol groups are protonated (Si–OH_2_^+^) and the resulting positively charged oxide surface hardly interacts with cations such as Ni^2+^.^20^ Liu et al.^21^ for the first time showed one-pot synthesis of silica-supported Ni catalysts with similar characteristics to that of Ni-KIT-6 with nanocrystals or amorphous nickel oxide species highly dispersed into the pore walls. The studied material showed enhanced stability in DRM compared to the counterpart prepared by conventional impregnation, with carbon deposition hardly detected by TGA and TEM after a 5 h test. The authors, however, did not disclose specifics of their preparation method, either the type of the utilized base solution for the pH adjustment or the precise pH value (only stated to be between 8 and 12). Yet, nickel incorporation into the KIT-6 wall structure was detrimental for textural properties of the resulting materials. This study confirms that elevated pH values are critical to achieving one-pot synthesis of Ni-KIT-6-like silicas.

Metal phyllosilicates have attracted considerable attention in recent catalysis studies due to their unique lamellar structure offering enhanced MSI, small metal domain size characterized with high dispersion, and remarkable thermal stability.^22^ Ni phyllosilicates (Ni-PS) consist of tetrahedral layers of SiO_4_ (Si–O–Si) and octahedral layers of Ni­(II) (Ni coordinated to oxygen atoms or hydroxyl groups, Si–O–Ni–O­(OH)).^23^ Ni-PS typically exists in two structural forms: (i) Ni_3_Si_2_O_5_(OH)_4_ composed of one Si–O–Si tetrahedral sheet bonded to one Si–O–Ni–O­(OH) octahedral sheet with the ratio of 1:1, exhibiting the nepouite structure, or (ii) Ni_3_Si_4_O_10_(OH)_2_ that consists of one Si–O–Ni–O­(OH) octahedral sheet sandwiched between two Si–O–Si tetrahedral sheets, resembling the talc structure.^24,25^ Sivaiah et al.^26^ investigated both types of nickel phyllosilicates as the catalyst precursor in dry reforming of methane. The authors reported that the 2:1 type led to an improved thermal stability with Ni phyllosilicate existing even at high reduction temperatures, being a key factor of high catalytic activity in DRM. The 1:1 structure collapsed upon reduction in the flow of H_2_ at 700 °C.

In the existing literature, Ni phyllosilicate (1:1) formation has not been addressed in the context of KIT-6. Under alkaline conditions, the pH value significantly affects polycondensation between Ni­(OH)_2_-derived species and surface silanol groups (Si–OH) on the SiO_2_ framework. In the systems that favor the 1:1 phyllosilicate structure, the condensation leads primarily to Si–O–Ni–OH, rather than 2:1 Si–O–Ni–O–Si. As described by Zhang et al.,^24^ a representative step of this process can be expressed as (eq 7):

$$\mathrm{Ni}{\left(\mathrm{OH}\right)}_{2}+\mathrm{Si}‐\mathrm{OH}\to \mathrm{Si}‐O‐\mathrm{Ni}‐\mathrm{OH}+H_{2}O$$ 7

Moreover, as far as we surveyed, the nature of 1:1 Ni phyllosilicates and their role in methane-rich dry reforming were not previously studied. In this paper, two catalysts were synthesized by different preparation methods, employing a one-pot synthesis approach (KIT-6/Ni onepot) and wet impregnation (KIT-6/Ni imp). The materials were characterized and tested in methane-rich dry reforming. We aimed at understanding the reduction course of nickel phyllosilicate present in KIT-6-like silica (KIT-6/Ni onepot catalyst) and its role in the catalytic process. The KIT-6/Ni imp catalyst was studied by us before and reported in our previous work.^15^

## 2 Experimental Section

### 2.1 Materials

Triblock copolymer Pluronic P123 (poly­(ethylene glycol)-*block*-poly­(propylene glycol)-*block*-poly­(ethylene glycol)), hydrochloric acid (HCl, 37% w/w in H_2_O), n-butanol (CH_3_(CH_2_)_3_OH), tetraethyl orthosilicate (Si­(OC_2_H_5_)_4_), and nickel­(II) nitrate hexahydrate (Ni­(NO_3_)_2_·6H_2_O) were obtained from Sigma-Aldrich. Ammonia solution (NH_4_OH, 25% w/w in H_2_O) was purchased from Supelco. All reagents were used without further purification.

### 2.2 Catalyst Preparation

The KIT-6/Ni onepot catalyst was synthesized by combining a hydrothermal treatment with the ammonia evaporation method. An aqueous solution was prepared by dissolving 4 g of triblock copolymer Pluronic P123 in 7.47 g of HCl (37% w/w in H_2_O) and water (144 mL) under vigorous stirring for 5 h. The solution was gradually heated to 35 °C under mechanical stirring; afterward, 10 mL of ethanol-based solution of Ni­(NO_3_)_2_·6H_2_O (0.061 mol) was added dropwise. Two hours later, n-butanol (4 g) was added, followed 1 h later by tetraethyl orthosilicate (TEOS, 8.6 g) under stirring at 35 °C. The resulting solution was aged for 20 h. The pH was further adjusted to 9 by dropwise addition of ammonia solution (25% (w/w) in H_2_O). A light-blue solution was obtained, which was left open for 2 h and then closed for an additional 2 h of aging. The aged solution was transferred into a Teflon bottle for hydrothermal treatment at 100 °C for 24 h under static conditions. The precipitate was recovered by filtration without washing. The wet paste was placed on a watch glass and dried at 70 °C overnight and further calcined at 600 °C for 6 h. The synthesis procedure of the KIT-6/Ni onepot catalyst is illustrated in Scheme S1.

For comparison, the KIT-6 support was synthesized at pH 9 using ammonia solution (25% w/w in H_2_O), following the same steps as for the KIT-6/Ni onepot, excluding the addition of the nickel precursor.

A conventional wet impregnation method was used to synthesize KIT-6/Ni imp. In short, Ni­(NO_3_)_2_·6H_2_O was dissolved in 80 mL of deionized water. The KIT-6 support, synthesized according to a previously reported procedure,^15^ was subsequently added and vigorously stirred for 24 h. The solution was transferred to a rotary evaporator at a 110 rpm rotating speed and a water bath set at 60 °C. Afterward, the product was dried overnight at 70 °C and calcined at 600 °C for 6 h.

### 2.3 Characterization Methods

Elemental composition of the studied catalysts was determined by ICP-OES (5110 Agilent VDV) after a two-step acid mineralization and microwave digestion (Anton-Paar Multiwave Pro). The samples were diluted in a mixture of 4 mL of HNO_3_ (>68%), 3 mL of HCl (34–37%), and 1 mL of HF (47–51%), followed by treatment with 3 mL of H_3_BO_3_ (>68%) and 5 mL of HCl (34–37%). All acid concentrations are given as weight percent (w/w) in aqueous solution.

N_2_ adsorption–desorption was used to measure the textural properties of the calcined samples. Around 50 mg of each sample was degassed at 300 °C for 3 h in a VacPrep 061 Degasser before transferring to a Micromeritics TriStar II 3020 Surface Area and Porosity Analyzer. Specific surface areas and pore volumes were calculated using, respectively, BET and BJH (desorption) methods at the temperature of liquid nitrogen (−196 °C). The pore sizes were corrected employing the Kruk–Jaroniec–Sayari (KJS) geometrical scheme.

The small-angle X-ray scattering (SAXS) patterns were acquired on a Bruker D8 instrument with Cu Kα radiation (λ = 0.15406 nm). The diffractograms were recorded in the range 0.6–5.0° with a step size of 0.01° (2θ) on a silicon sample holder at room temperature.

XRD was carried out at room temperature on uncalcined samples and on materials collected after 50 h of catalytic test. Ex situ measurements on a Bruker D8 instrument using Cu Kα radiation (λ = 0.15406 nm) were conducted. The diffractograms were recorded in the range of 7–90° (2θ) on a silicon sample holder.

In situ XAS-XRD measurements during reduction and methane-rich dry reforming were conducted at the BM31 (SNBL) of the European Synchrotron Radiation Facility (ESRF) in France. The sample was stabilized between two plugs of quartz wool in a capillary reactor (Hilgenberg GmbH) with a wall thickness of 0.01 mm and an outer diameter of 1.0 mm. The beam size (unfocused beam) at BM31 is around 4.0 × 0.3 mm for XAS, but with a larger vertical slit opening for XRD (4.0 × 1.2 mm when using a 1.0 mm capillary). Reduction was carried out in a mixture of 4% H_2_/He, following the same procedure as for TPR-H_2_ (Figure S1). For the methane-rich dry reforming over KIT-6/Ni onepot and KIT-6/Ni imp catalysts, 3.0 and 3.5 mg were used, respectively, with the size fraction between 53 and 90 μg. The setup was supplemented by a gas blower to control the reactor temperature. The detailed experimental procedure is presented in Figure S2. The XAS spectra were collected in the transmission mode at the Ni K-edge. The XRD data were collected alone or together with XAS, using a DEXELA CMOS 2D. The incident beam for XRD was set at a wavelength of 0.0338 nm. The XAS-XRD measurements were conducted in a continuous loop, using a macro programmed to acquire XANES and XRD data within 1 min 34 s, while EXAFS acquisition required 3 min 20 s. The Scherrer equation was used to estimate the crystallite size of metallic nickel, assuming Scherrer’s constant K = 0.94 for spherical nickel crystallites with cubic symmetry.^15^

Temperature-programmed reduction in H_2_ was performed on a BELCAT-M (BEL Japan) analyzer. The sample (ca. 30 mg) was pretreated in Ar at 300 °C for 1 h. TPR-H_2_ experiments were performed by heating the sample from room temperature to 750 °C (10 °C/min) in a mixture of 5% H_2_/Ar and then keeping it at 750 °C for 90 min (Figure S1).

High-resolution transmission electron microscopy (HRTEM) micrographs were acquired on a JEOL JEM-2100 Plus instrument operating at 200 kV. The catalyst powder was highly dispersed in ethanol with vigorous ultrasonication. Then, a drop of suspension was dripped onto a copper grid.

The hydrogen chemisorption on the catalysts was obtained by using a Micromeritics ASAP2020. The catalyst was reduced in a pure H_2_ flow at 750 °C (10 °C/min) and held at 750 °C for 90 min. The uptake of H_2_ was calculated from the isotherms collected at 40 °C. The dispersion of Ni was estimated assuming H/Ni = 1.^10^

Infrared spectra were acquired on a 1.5 cm diameter self-supporting pellet of about 15 mg of powder pressed at 2 tones. Prior to the CO adsorption, the sample was outgassed (residual pressure 10^–4^ mbar) under heating up to 650 °C and then reduced under 5 mbar of H_2_ for 90 min. CO doses were recorded at room temperature using a Nicolet iZ10 spectrometer with an MCT detector cooled by liquid nitrogen by accumulating 64 scans at a 4 cm^–1^ resolution.

Thermogravimetric analysis was performed on a TGA Q500 instrument to quantify carbon formation. The initial step involved setting the temperature at 120 °C for 30 min in a flow of synthetic air (20 mL/min). Subsequently, the temperature was gradually increased from 120 to 900 °C (5 °C/min) under a continuous flow of synthetic air (20 mL/min).

### 2.4 Catalytic Tests in Methane-Rich Dry Reforming

Catalytic tests were performed at atmospheric pressure in a fixed-bed U-shaped quartz reactor filled with 50 mg of the catalyst (53–90 μg fraction) placed between two quartz wool plugs. A thermocouple of type K was used and placed outside the catalytic bed, i.e., not in direct contact with the powder catalyst and gaseous reactants. Samples were reduced in a mixture of 5% H_2_/Ar from room temperature to 750 °C (10 °C/min), held for 90 min, purged with Ar, and cooled to 700 °C. The reaction was conducted with a total flow of 100 mL/min: 53 mL/min CH_4_, 30 mL/min CO_2_, 17 mL/min Ar (WHSV= 120,000 mL/(h·g_cat_)), corresponding to the CH_4_/CO_2_ ratio of 1.8. The conversions of methane and carbon dioxide were intentionally kept below equilibrium to accurately assess the real deactivation trends. The effluent gas (excluding H_2_O) was analyzed by gas chromatography (490 Varian Micro-GC).

## 3 Results and Discussion

### 3.1 Reducibility of the KIT-6/Ni Catalysts

XAS measurements were carried out to obtain information on the nickel environment at the Ni K-edge. In Figure 1a, the XANES profile of KIT-6/Ni imp is similar to that of the NiO standard with a white line at ca. 8350 eV. The spectrum belonging to KIT-6/Ni onepot is different from those of the reference compounds, although some similarities to the Ni­(OH)_2_ standard can be found. Among the noticeable differences, a XANES pre-edge shift to lower energy values is visible (Figure S5). While the KIT-6/Ni imp catalyst exhibited the typical reduction of NiO to Ni^0^ (Figure 1b), the KIT-6/Ni onepot sample showed distinct reduction behavior (Figure S6).

**Figure 1:**
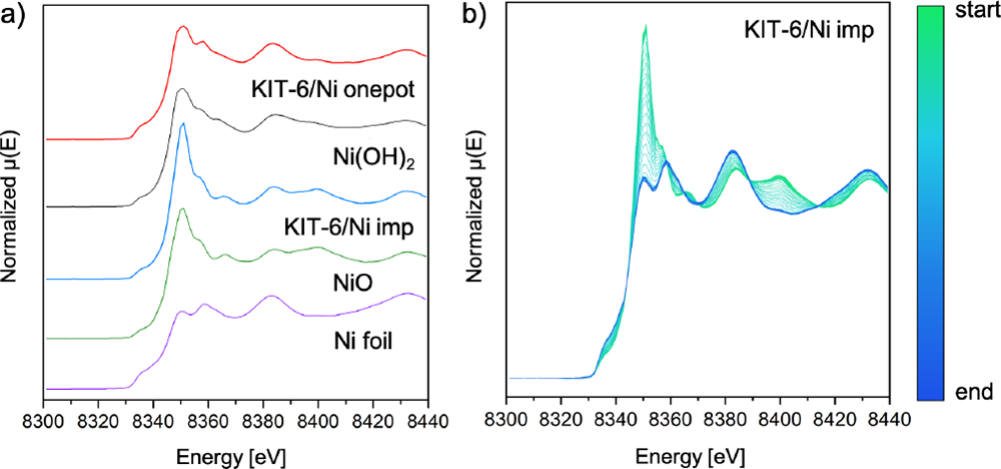
Ni K-edge XANES spectra for the calcined KIT-6/Ni onepot and KIT-6/Ni imp catalysts and the standards: Ni foil, NiO, and Ni­(OH)_2_ (a). XANES spectra collected in situ during reduction of KIT-6/Ni imp (b). Reduction conditions: *T* = 750 °C for 90 min, heating rate 10 °C/min, 4% H_2_/He feed with a total flow of 7.0 mL/min for KIT-6/Ni imp.

The detailed changes of XANES during the reduction are shown in Figure 2a–k to determine the evolution of the Ni^2+^ environment in the Ni_3_Si_2_O_5_(OH)_4_-containing material. The first spectra collected within the temperature range of 50–150 °C did not show significant changes (Figure 2a). Once the temperature reached 160 °C, the intensity of the white line increased, however, without a shift in energy (Figure 2b). At this temperature, the sample primarily undergoes dehydration of physiosorbed water, and the collected results can be interpreted as a distortion of the coordination of Ni, most likely involving H_2_O.^27^ This is in good agreement with changes in the MS signal assigned to *m*/*z* = 18 (H_2_O) (Figure S7). The subsequently collected XANES spectra revealed a progressive reduction at higher temperatures (Figure 2c–f). According to in situ XRD, at the temperature of 457 °C (Figure 3a), initial reduction of Ni^2+^ to Ni^0^ occurred and nanoparticles started to form, most likely within the silica matrix. At 587, 652, and finally at 750 °C (Figure 3a), the XRD reflections of metallic nickel became more pronounced, at the expense of the Ni_3_Si_2_O_5_(OH)_4_ phase. The LCF (linear combination fitting) of XANES spectra showed that Ni was only partially reduced to metallic nickel (Figure S8). The unreduced nickel remained in the form of Ni-PS, still resembling a separate phase with a very low crystallinity (Figure 3b). The sample was subsequently held at 750 °C for the next 90 min (Figure 2g–k). The first minutes did not show differences in XANES spectra. After ca. 9 min of the treatment, an increase in intensity of the white line became more pronounced compared to dehydration recorded at lower temperatures (Figure 2h vs Figure 2b). This change was also visible after about 10 min in the recorded XRD patterns (Figure 3b). Ni_3_Si_2_O_5_(OH)_4_ appeared as a more distinct crystalline phase. One of the hypotheses for this observation may be the formation of water during the reduction of nickel species that are reduced only after several minutes at 750 °C, as illustrated in the TPR-H_2_ profile (Figure 2l). Possibly, hydroxylation of nickel phyllosilicate ensued, regardless of the high temperature and reducing conditions. Another hypothesis is that the loss of hydroxyl groups, accompanied by water release, resulted from the thermal Ni-PS decomposition. According to Sivaiah et al.,^28^ this process can occur from 450 °C even up to 800 °C. In our case, dehydroxylation could cause a humid environment potentially leading to the reconstruction of Ni-PS. The LCF fraction of the hydroxylated phase (in our case Ni­(OH)_2_ used as a standard) increased from 0.25 to 0.67 (Figure S8). During the remaining time of the isothermal reduction, the fraction of metallic nickel in the sample slowly increased. Ultimately, the sample was less reduced compared to the state when it first reached 750 °C (Figure 2k vs Figure 2f). Our spectra (Figure 2) are in good agreement with the collected XANES of the reduced Ni-PS-containing SBA-15/Ni described in the study of Hongmanorom et al.^29^

**Figure 2:**
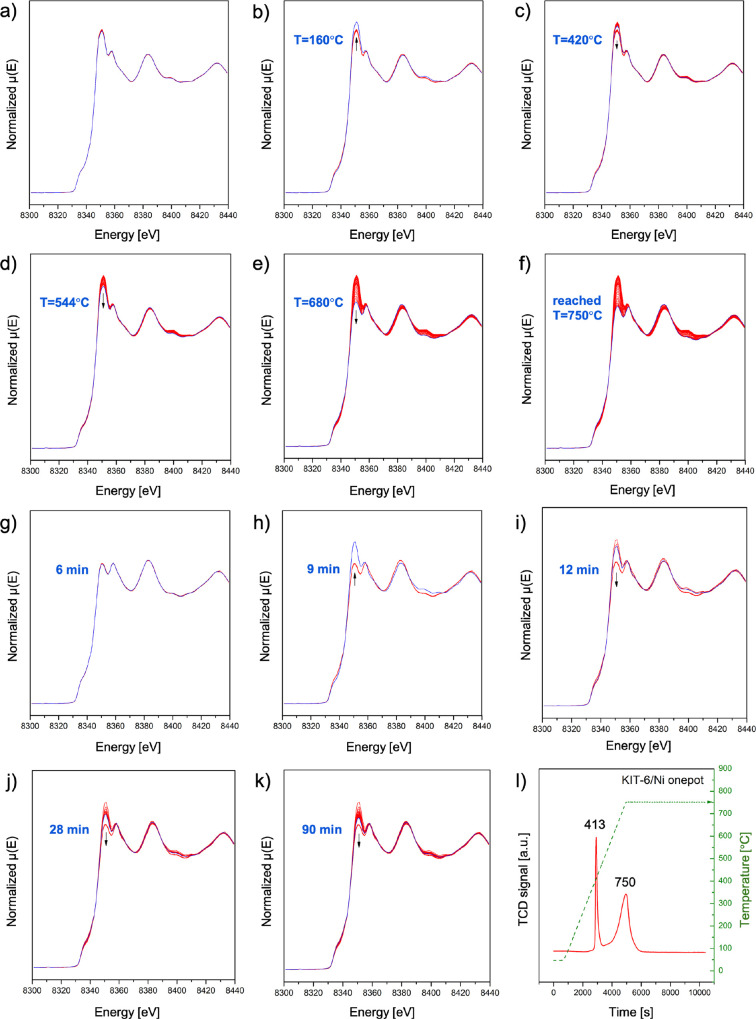
Ni K-edge XANES spectra of the KIT-6/Ni onepot catalyst collected in situ during reduction in a mixture of 4% H_2_/He, i.e., ramping up from 50 to 750 °C (a–f) and keeping the sample for 90 min at 750 °C (g–k). TPR-H_2_ profile of the KIT-6/Ni onepot (l). Reduction conditions for spectra (a–k): *T* = 750 °C for 90 min, heating rate 10 °C/min, 4% H_2_/He feed with total flows of 6.0 mL/min for KIT-6/Ni onepot and 7.0 mL/min for KIT-6/Ni imp.

**Figure 3:**
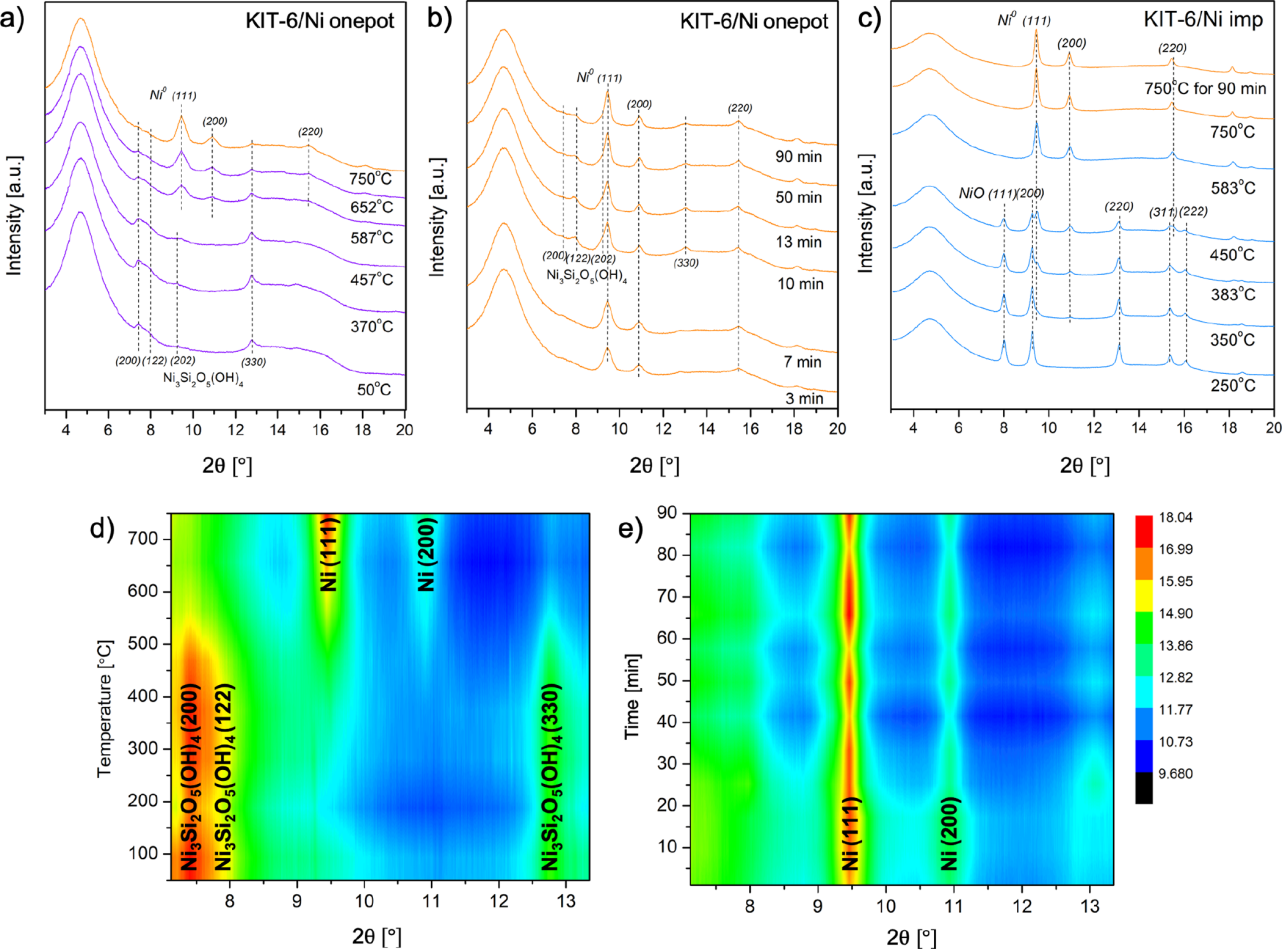
XRD patterns acquired in situ (λ = 0.0338 nm): (a) KIT-6/Ni onepot recorded during the reduction from 50 to 750 °C and (b) when the catalyst was held for 90 min at 750 °C. (c) XRD patterns of KIT-6/Ni imp during the entire course of reduction from 50 to 750 °C. (d) Contour plots of the KIT-6/Ni onepot during reduction as a function of temperature (heating rate 10 °C/min, up to 750 °C), (e) followed by isothermal reduction for 90 min. The most prominent diffraction peaks are labeled. Reduction conditions: *T* = 750 °C for 90 min, heating rate 10 °C/min, 4% H_2_/He feed with total flows of 6.0 mL/min for KIT-6/Ni onepot and 7.0 mL/min for KIT-6/Ni imp.

To supplement the XRD results, the evolution of phases recorded during the course of reduction is presented in the contour plots depicted in Figure 3d,e. As illustrated, the presence of nickel phyllosilicate is the most pronounced until ca. 520 °C and decreased at higher temperatures as a consequence of its partial reduction to metallic nickel, reflected in the appearing Ni(111) and Ni(200) peaks. The Ni(111) reflection is the most intense and dominates during the isothermal reduction. Still, some contribution of Ni-PS can be observed throughout the reduction course.

From the in situ collected diffractograms, it was possible to estimate the average size of Ni^0^ crystallites, which was calculated from the most intense peak not overlapping with other nickel species (Table 1). For the samples reduced at 750 °C, the crystallite sizes were as follows: KIT-6/Ni onepot 4.3 ± 0.1 nm, KIT-6/Ni imp 10.2 ± 0.2 nm, while the final crystallite sizes after the entire reduction course were KIT-6/Ni onepot 6.6 ± 0.1 nm, KIT-6/Ni imp 10.1 ± 0.1 nm. The presence of sintering can be excluded in the case of KIT-6/Ni imp since no significant increase in Ni crystallite sizes was observed. We decided to take a closer look at the reduction of the KIT-6/Ni onepot catalyst. A detailed analysis of the data collected under isothermal conditions (750 °C for 90 min) was performed to exclude the influence of thermal expansion of the material’s lattice with increasing temperature. The Ni crystallite size was estimated for the selected X-ray diffractograms and is illustrated in Figure 4. The first minutes of reduction showed crystallite sizes in the range of 4–4.5 nm. After ca. 10 min, a significant increase in crystallite sizes was observed, which is consistent with the changes in the oxidation state recorded in XANES due to water release. An increase in the intensity of the (122) reflection compared to the (200) plane was observed, suggesting possible changes in crystallographic orientation (Figures 3b and S9). Additionally, the pronounced shift of the (330) reflection toward higher 2θ values indicates that structural reconstruction in the presence of water could lead to lattice contraction.^30^ The water formation was confirmed by mass spectrometry (Figure S7). Two main temperature zones can be seen in which the MS signal increased for *m*/*z* = 18, i.e., 110–220 and 610–750 °C. The time factor deserves an additional consideration, as water vapor is likely formed before any detectable changes appear in the XANES spectra.

**Table 1:** Nickel Crystallite Size, Dispersion, and Reducibility

catalyst	Ni^0^ crystallite size [nm]^a^	Ni^0^ crystallite size [nm]^b^	Ni^0^ crystallite size [nm]^c^	Ni^0^ crystallite size [nm]^d^	H_2_ uptake [μmol/g]^e^	degree of reduction [%]^e^	Ni dispersion [%]	Ni crystallite size [nm]
KIT-6/Ni imp	10.2 ± 0.2	10.1 ± 0.1	10.2 ± 0.1	10.3 ± 0.1	1.03	97.8	8.2^f^ (7.8)^g^	12^h^(13)^i^
KIT-6/Ni onepot	4.3 ± 0.1	6.6 ± 0.1	6.7 ± 0.1	6.5 ± 0.2	0.77	75.9	13.6^f^ (9.5)^g^	7^h^(11)^i^

a Reached 750 °C during reduction in a mixture of 4% H_2_/He, calculated from XAS-XRD data utilizing the diffraction peak at 2θ = 10.9°.

b After 90 min at 750 °C during reduction in a mixture of 4% H_2_/He, calculated from XAS-XRD data utilizing the diffraction peak at 2θ = 10.9°.

c Start of the catalytic test at 700 °C, calculated from XAS-XRD data utilizing the diffraction peak at 2θ = 10.9°.

d End of the catalytic test at 700 °C, calculated from XAS-XRD data utilizing the diffraction peak at 2θ = 10.9°.

e Calculated based on the area under the TPR-H_2_ curve, with Ni loading given by ICP-OES, and assuming that the theoretical amount of H_2_ required for the overall reduction of Ni species is 0.85 mmol/g for 5 wt % of metal loading,^31^ our catalysts contain ca. 6 wt % Ni.

f Nickel dispersion (D) calculated based on H_2_ chemisorption for the calcined sample using hydrogen chemisorption data and corrected by the degree of reduction of Ni^2+^ species.

g Values in parentheses correspond to dispersion calculated by HRTEM (eq S1).

h Crystallite size of the reduced sample, assuming spherical metal crystallites of uniform diameter, calculated as Ni crystallite size = 97.1/D.^32^

i Average crystallite size estimated by statistical analysis of HRTEM micrographs.

**Figure 4:**
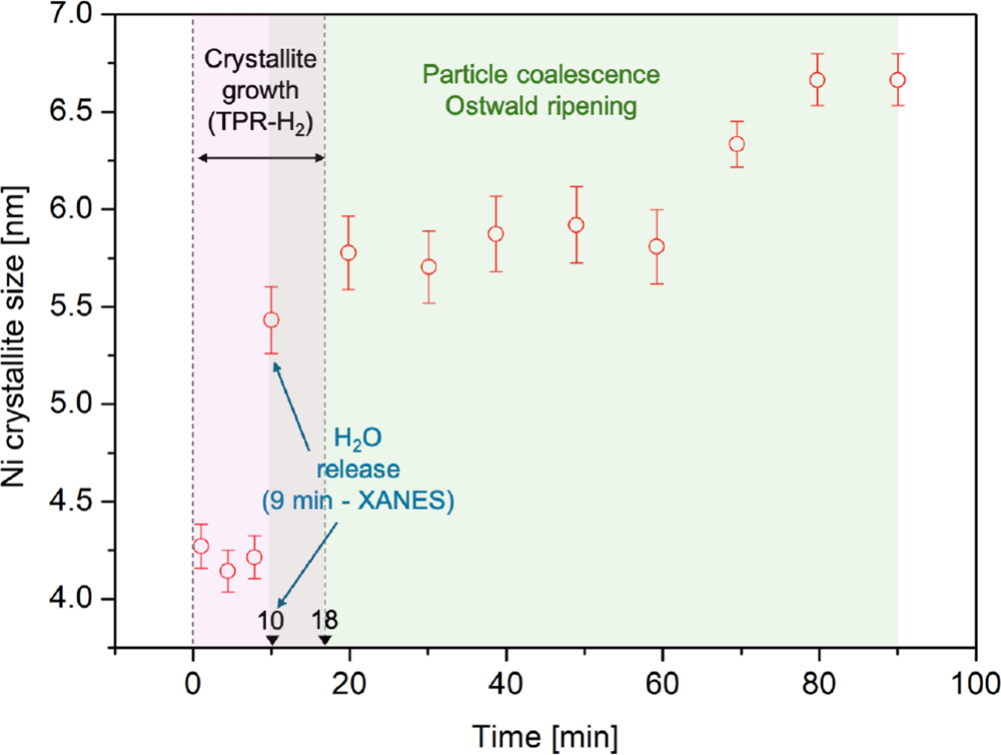
Changes in the nickel crystallite size as a function of time registered during the isothermal reduction at 750 °C for 90 min (KIT-6/Ni onepot). The crystallite sizes were estimated using XRD patterns collected in situ.

Since the alterations in the catalyst structure detected by XANES and XRD appeared after ca. 9–10 min, and the corresponding change in the MS (peak in the signal *m*/*z* = 18) occurred after 11 min, one can think of a specific water buildup or just a delay in the signal registration. Subsequent growth of Ni crystallites resulted in an average size of ca. 6.6 nm. In general, the changes in the crystallite size can be divided into two stages:

1. Nickel crystallite growth originating from the reduction of the remaining fraction of phyllosilicate at 750 °C, which can be understood from Figure 2l, and
2. nickel agglomeration caused by coalescence, growth, or shrinkage of the small particles and their disappearance by Ostwald ripening type phenomena.

The second assumption follows a recent study of Turner et al.^33^ The authors performed in situ TEM investigating the influence of water vapor on the reduction (0.7% H_2_O/5% H_2_/ 94.3% Ar) of nickel phyllosilicates at a temperature of 700 °C. It was observed that H_2_O led to faster particle growth and more frequent Ostwald ripening and coalescence events. Additionally, water vapor allowed larger, less dense particles to shrink. The presence of water clearly affected the degree of reduction since more nickel nuclei could be formed under hydrogen flow in the dry feed. A similar effect could have contributed to the incomplete reducibility recorded in our KIT-6/Ni onepot sample.

TPR-H_2_ was carried out to investigate reducibility of nickel containing catalysts (Figure S10). The impregnated Ni catalyst revealed overlapping reduction peaks in the temperature region of 250–650 °C originating from either NiO weakly interacting with the surface or bulk NiO and reduction of NiO with strengthened MSI. KIT-6/Ni onepot catalyst showed a single reduction peak at 413 °C and a broad reduction peak at higher temperatures from 500 to 750 °C. Considering the presence of Ni phyllosilicate, and the absence of NiO in the calcined KIT-6/Ni onepot, both registered peaks were attributed to the reduction of Ni^2+^ present in nickel phyllosilicate with medium (413 °C) and strengthened interactions (500–750 °C) between nickel species and the silica support.^22^ The recorded changes match well with the in situ XRD results (Figure 3). The TPR-H_2_ results suggested a complete reduction of nickel species to metallic nickel for both catalysts. Nevertheless, considering the XANES results, Ni^2+^ species in the Ni-PS-containing catalyst seemed to be highly stable. According to Kuhaudomlap et al.^22^ and Zhang et al.,^24^ Ni-PS tend to reduce at temperatures up to 800 °C. In our study, the reduction behavior was not investigated at temperatures higher than 750 °C. The calculated H_2_ uptake and the degree of reduction are listed in Table 1. While KIT-6/Ni imp exhibited a reduction degree close to 100%, the nickel cationic species in the Ni-PS-containing catalyst were stabilized, resulting in a reduction degree of 75.9% for the KIT-6/Ni onepot. Clearly, this difference from the estimated XANES LCF fraction may originate from the fact that Ni­(OH)_2_ was used as the reference, which may not fully reflect the real XANES pattern of Ni_3_Si_2_O_5_(OH)_4_.

High-resolution microscopy provided crucial evidence of the presence of an ordered mesoporous architecture in the KIT-6 support. While reduced KIT-6/Ni imp represents typical cubic mesopores (Figure 5a–c), KIT-6/Ni onepot shows disordered mesopores of various shapes and the absence of *Ia3 ®d* construction (Figure 5d–f). This is consistent with the small-angle XRD analysis (Figure S3b). Moreover, based on the N_2_ sorption results (Figure S3a), the H3 hysteresis loop indicates a transition toward slit-like and irregular porosity rather than the uniformly interconnected pore architecture characteristic of pristine KIT-6. Wang et al.^23^ investigated Ni/SiO_2_ catalysts containing Ni phyllosilicates. The authors found in their HRTEM micrographs homogeneous Ni nanoparticles entrapped in Ni-PS, which restricted the agglomeration of nickel species during reduction. The Ni particles were tightly surrounded by fibrous Ni-PS after reduction, which is consistent with micrographs of the sample (Figure 5e,f). Similar structural construction was also found in the study of Turner et al.^33^ After impregnation with Ni, the 3D-mesoporous channels were still regular and uniform, suggesting that the addition of metal species did not destroy the well-defined channels. The nickel particles were larger for the KIT-6/Ni imp than for the KIT-6/Ni onepot sample (Figure 5c vs Figure 5f).

**Figure 5:**
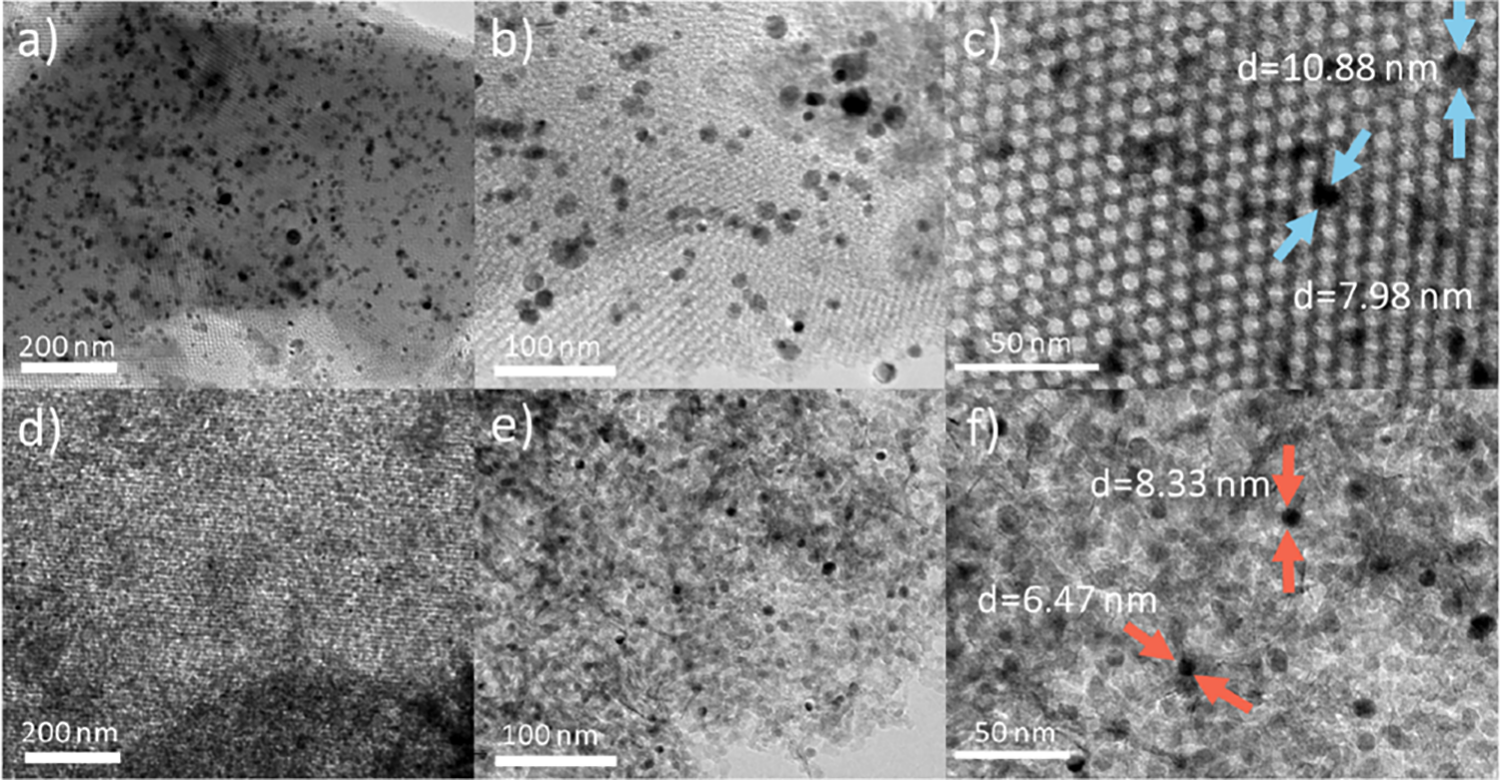
HRTEM of reduced KIT-6/Ni imp (a–c) and KIT-6/Ni onepot (d–f).

Hydrogen chemisorption was employed to estimate the capacity responsible for the number of exposed metal sites corresponding to the catalytically active sites. The dispersion values, corrected by the degree of reduction, were 13.6% for KIT-6/Ni onepot and 8.2% for KIT-6/Ni imp (Table 1). This follows a trend similar to that reported in the literature.^29^ It should be highlighted that the large number of active sites is due to relatively abundant free Ni species resulting from Ni phyllosilicate instead of NiO.

### 3.2 Surface Species on the KIT-6/Ni Catalyst Surface

IR analysis during pretreatment clearly indicated silanol condensation, shown by a gradual disappearance of a broad band with increasing temperature and the emergence of a sharp peak at 3745 cm^–1^ for both samples (Figure S14). This peak is generally associated with the presence of isolated OH groups. In the impregnated sample, this peak split into two, suggesting the coexistence of additional OH species, specifically an intermediate form, i.e., vicinal silanol groups that are weakly interacting with hydrogen. With the increase of the temperature, isolated and vicinal OH groups are present in nearly equal contributions. Upon completion of the pretreatment, isolated OH groups prevail as a result of continued silanol condensation, and in both samples, OH groups involved in hydrogen bonding are no longer detected. In the sample synthesized via one-pot approach, an additional peak at 3625 cm^–1^ was observed, which was significantly affected by thermal treatment. This band can be associated with the ν_OH_ vibration related to the nickel hydroxide and/or the octahedral-coordinated Ni­(II) in the sheet of 1:1 nickel phyllosilicate.^34^ This observation aligns well with the XAS-XRD results, indicating a partial loss of the crystalline phase of Ni-PS at elevated temperatures, e.g., 650 °C. Additionally, during the first minutes of pretreatment, the removal of molecularly adsorbed water was detected, as evidenced by a stronger band at 1650 cm^–1^. This band quickly disappeared upon heating and outgassing of the sample and could be related to the oxidation changes similar to those detected by XANES (Figure 2b). Notably, this band was not observed in the impregnated sample; however, its absence does not rule out the occurrence of similar behavior. The ability to detect such features depends on the initial conditions of the IR experiment and the timing of data acquisition.

Furthermore, we employed CO as a probe molecule to explore the adsorption modes on nickel active sites (Figure 6a,b). A significant contribution of linear (or terminal) CO adsorbed on the nickel atom (Ni^0^–CO) was observed at 2045 cm^–1^ for both catalysts. Its intensity increased with an increasing equilibrium pressure. Moreover, the presence of bridged Ni^0^_2_–CO (1950–1900 cm^–1^), as well as di- and tricarbonyl species (2080–2050 cm^–1^), could be detected.^1,35^ In the case of the KIT-6/Ni onepot catalyst, more possibilities of CO binding were observed. Additional bands were recorded above 2100 cm^–1^, which indicates the presence of nickel in the ionic state. According to the literature,^36–39^ the stretching modes of nickel carbonyls appear in distinct spectral regions: 2220–2180 cm^–1^ for Ni^2+^–CO (in our study: 2195 and 2186 cm^–1^) and 2160–2110 cm^–1^ for Ni^+^–CO (in our study: 2163, 2150, and 2110 cm^–1^). Bands observed at 2170 and 2121 cm^–1^ can be attributed to traces of gaseous CO.^36,40^

**Figure 6:**
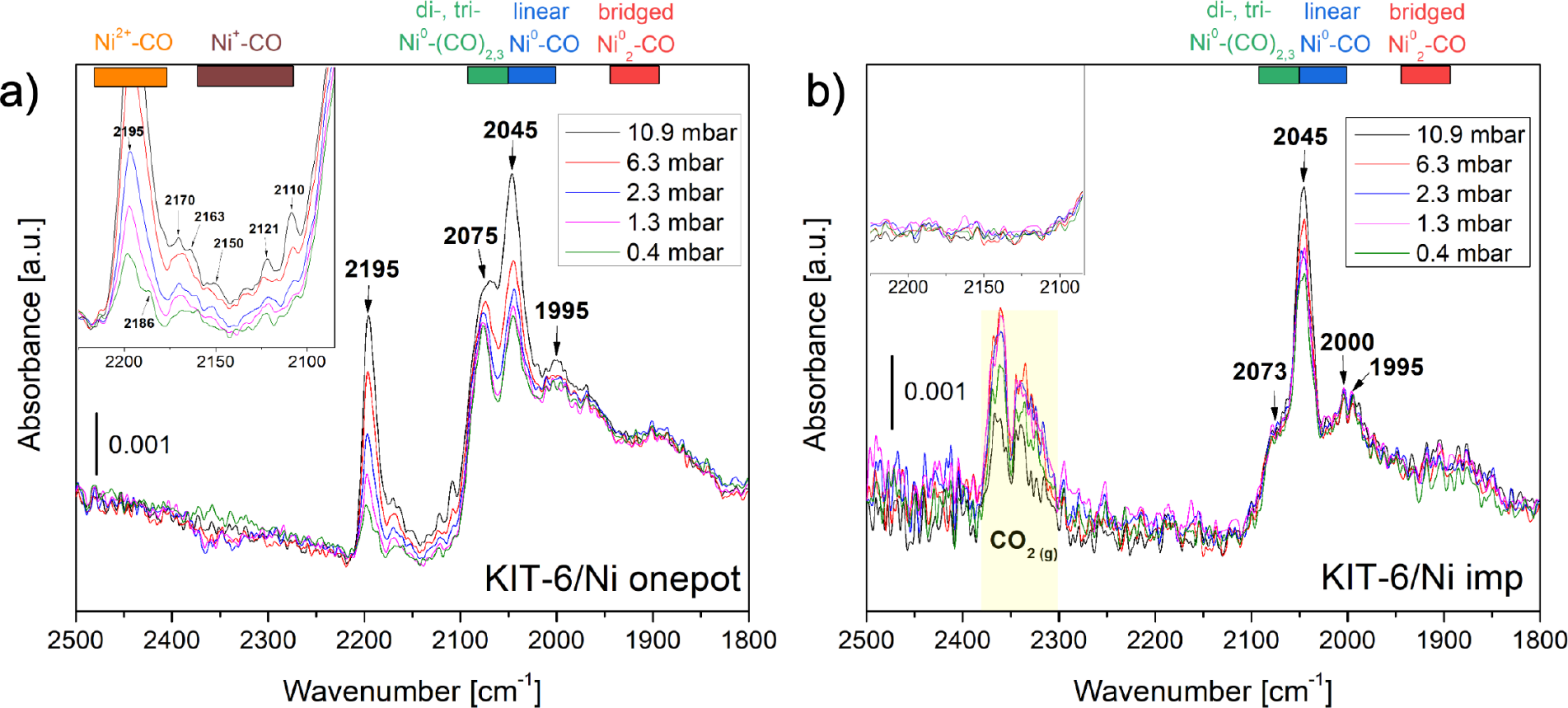
IR spectra of CO adsorption on (a) KIT-6/Ni onepot and (b) KIT-6/Ni imp catalysts. Pretreatment conditions: outgassing under 10^–4^ mbar up to 650 °C, followed by reduction under 5 mbar H_2_ for 90 min. CO adsorption was recorded at room temperature using various doses of CO.

The presence of CO_2_ in KIT-6/Ni imp is related to the Boudouard reaction (eq 3), which is a highly structure-sensitive process that occurs preferentially on step sites.^41,42^ For nickel catalysts, it ensues at room temperature, producing adsorbed CO_2_ able to react with the nucleophilic hydroxyl groups to form bicarbonate species, while carbonate-like species are produced through the interaction of CO_2_ with surface basic oxygen ions.^42^ For KIT-6/Ni onepot, neither CO_2_ nor bicarbonate/carbonate-like species were observed by IR spectroscopy, which shows that the one-pot synthesis approach led to suppression of the CO disproportionation. This behavior can be attributed to the used synthesis approach, which produces Ni species that are partially cationic and strongly stabilized within the silica matrix, thereby reducing the formation of extended metallic Ni step sites. Because the Boudouard reaction (eq 3) is known to be strongly site-selective at room temperature, the Ni ionic environments can effectively suppress the reaction. Other plausible explanations include the presence of edge/kink sites, as a smaller particle size observed for this catalyst generally increases the fraction of such sites. The size-dependent effect of the metallic particles with the FCC structure was widely reported for surface sensitive reactions.^43^

### 3.3 Catalytic Activity in Methane-Rich Dry Reforming

In situ XAS-XRD experiments are presented in Figure 7a–d. KIT-6/Ni onepot and KIT-6/Ni imp retained their oxidation state compared to that after reduction, which indicates that the catalyst deactivation may not be a consequence of a rapid oxidation, which would lead to the loss of the active phase.^10^ The former catalyst preserved its incomplete reduction degree. In situ XRD showed that the mixture of Ni phyllosilicate and Ni^0^ is stable under methane-rich dry reforming conditions (Figure 7c). No significant crystallite growth was registered for the metallic phase (Table 1). For KIT-6/Ni imp, a graphite peak in which intensity increased over time was recorded (Figure 7d). EXAFS spectra collected after the catalytic tests were compared with those acquired for the reduced samples (Figure S13). Fourier transform profiles at the Ni K-edge of the KIT-6/Ni imp and KIT-6/Ni onepot did not change significantly, indicating that the local order around Ni atoms was similar before and after reaction.

**Figure 7:**
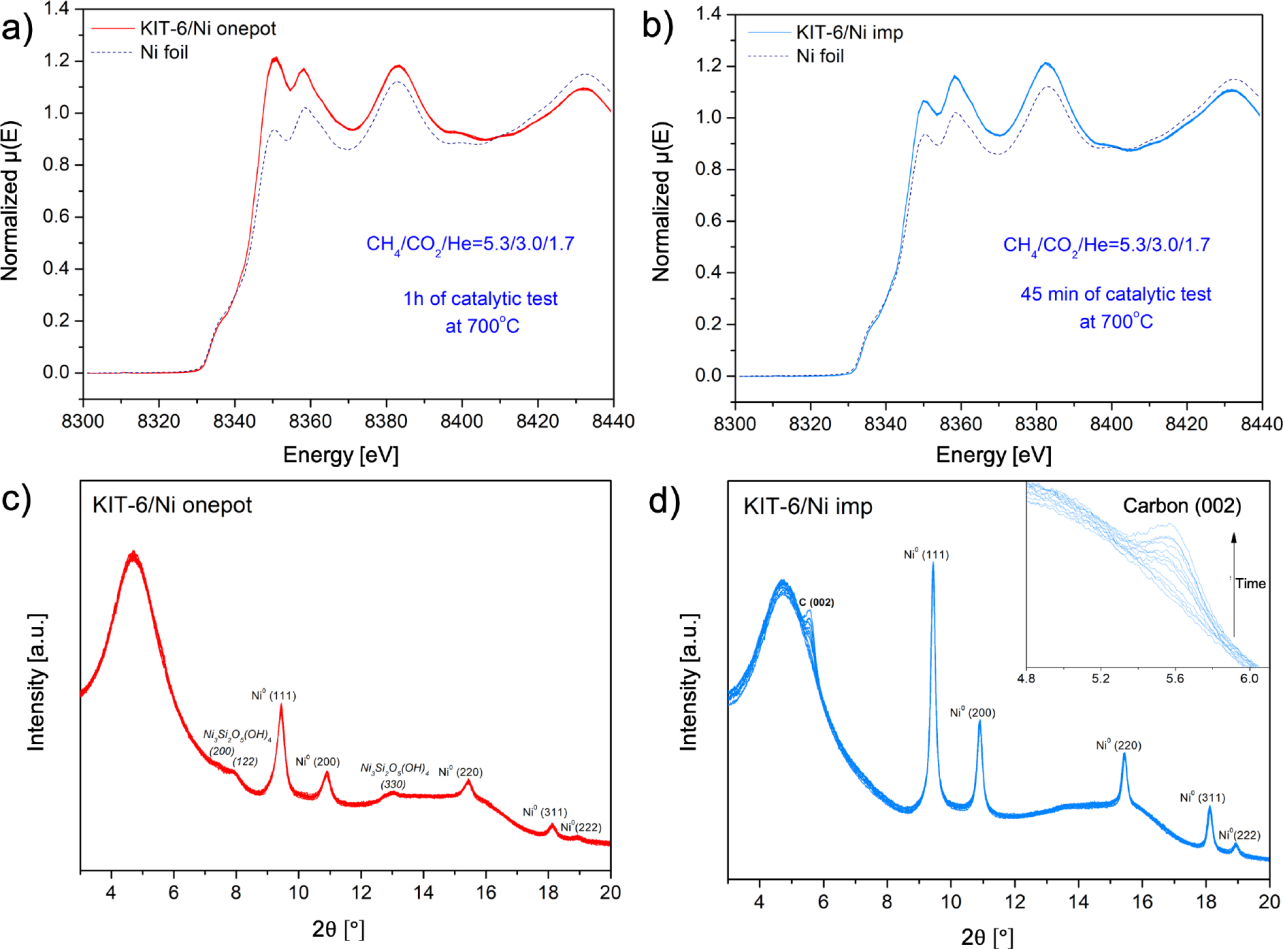
In situ XAS-XRD changes (a–d) for KIT-6/Ni onepot and KIT-6/Ni imp catalysts tested in methane-rich dry reforming. Reduction conditions: *T* = 750 °C for 90 min, heating rate 10 °C/min, 4% H_2_/He feed with total flows of 6.0 mL/min for KIT-6/Ni onepot and 7.0 mL/min for KIT-6/Ni imp. Reaction conditions: *T* = 700 °C for 1 h (KIT-6/Ni onepot) or 45 min (KIT-6/Ni imp), WHSV= 120,000 mL/(h·g_cat_), a gas mixture with a total flow of 6.0 mL/min for KIT-6/Ni onepot: 3.2 mL/min CH_4_, 1.8 mL/min CO_2_, and 1.0 mL/min He, and a total flow of 7.0 mL/min for KIT-6/Ni imp: 3.7 mL/min CH_4_, 2.1 mL/min CO_2_, and 1.2 mL/min He.

KIT-6/Ni imp and KIT-6/Ni onepot catalysts were compared in catalytic tests carried out for 264 min, as shown in Figure 8. The second material showed remarkably stable catalytic behavior in terms of CO_2_ and CH_4_ conversions, as well as the H_2_/CO molar ratio, while KIT-6/Ni imp exhibited a declining trend (Figure 8a,b). This behavior is consistent with rapid graphitic carbon accumulation (observed in Figure 7d), resulting in a reduction in CH_4_ activation and diminished CO_2_-driven carbon oxidation. The calculated consumption rates of CH_4_ and CO_2_ are shown in Figure 8c. For the KIT-6/Ni onepot, both rates were stable during the experiment, with CO_2_ consumed at a slightly higher rate than CH_4_. This balance can imply that the KIT-6/Ni onepot catalyst is well-optimized for both carbon dioxide reduction (CO_2_ ⇌ CO + O) and methane decomposition (CH_4_ ⇌ C + 2H_2_).^33^ In contrast, the impregnated catalyst demonstrated more evident differences between the CH_4_ and CO_2_ consumption rates. This greater instability observed for the impregnated counterpart can indicate a carbon deposition or overproduction of undesired side products. Hence, using this indirect measure, a faster deactivation is expected for KIT-6/Ni imp. Although the CH_4_ consumption rate appeared to be considerably higher for KIT-6/Ni onepot, this catalyst showed more stable behavior over time compared to the impregnated sample, suggesting that methane plays a role in the one-pot catalyst. A plausible explanation can be a mechanism previously described by Gili et al.,^7^ in which first surface carbon, as a reactive intermediate, is formed (eq 2), followed by oxidation of C via reverse Boudouard reaction (inverse eq 3). The former becomes thermodynamically favored over the forward Boudouard reaction at temperatures above ca. 650 °C.^7^

**Figure 8:**
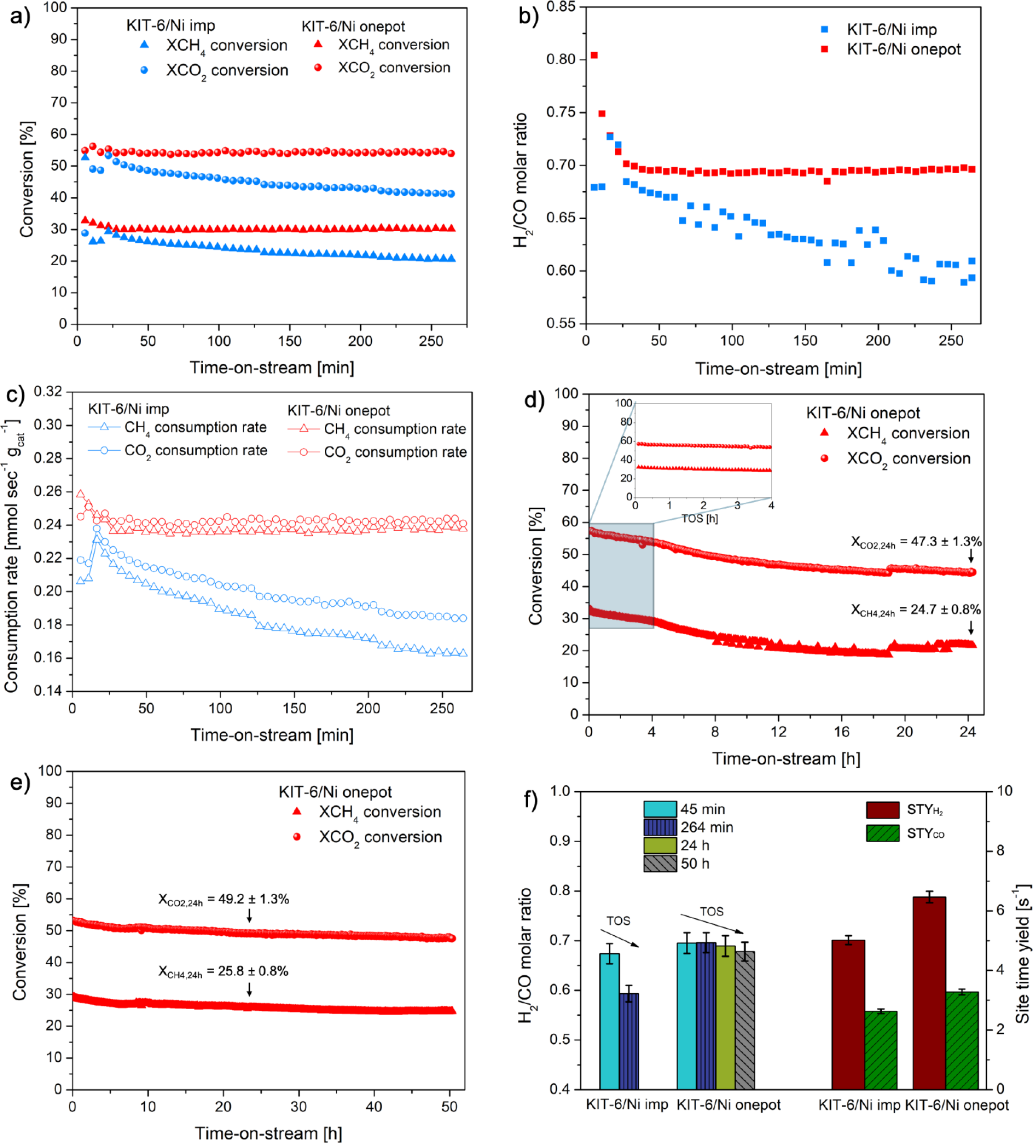
Catalytic tests in methane-rich dry reforming over KIT-6/Ni onepot and KIT-6/Ni imp. (a) CH_4_ and CO_2_ conversions recorded during a 264 min test. (b) H_2_/CO molar ratio as a function of time on stream. Reduction conditions: *T* = 750 °C for 90 min, heating rate 10 °C/min, 100 mL/min of total flow (5% H_2_/Ar). Reaction conditions: *T* = 700 °C, WHSV= 120,000 mL/(h·g_cat_) total flow of 100 mL/min: 53 mL/min CH_4_, 30 mL/min CO_2_, 17 mL/min Ar. (c) CH_4_ and CO_2_ consumption rates. (d) Long-term stability test carried out for 24 h time on stream. (e) Long-term stability test for 50 h time on stream. (f) Site time yield calculated after 45 min of catalytic tests, assuming Ni dispersion acquired from HRTEM, the weight fraction of nickel corrected by the accessible nickel (reducibility degree), and no change in the Ni crystallite size as monitored by in situ XRD.

Long-term stability for KIT-6/Ni onepot was evaluated in two catalytic tests for 24 h (Figure 8d) and 50 h (Figure 8e). The catalyst showed a stable trend in both reactions. Both independently conducted experiments confirmed the catalytic reproducibility of the tested catalyst. The estimated experimental error for all of the conducted tests is listed in Table S3. To get insight into the catalysts’ stability, both tested samples were characterized by two different techniques (24 h spent sample by HRTEM and 50 h spent sample by TGA) as described further in the text. Moreover, more dispersed Ni species resulted in a more stable catalytic performance during DRM, and this enhanced stability was correlated with higher STY values calculated after 45 min of the catalytic test (Figure 8f). This duration was chosen because in situ XAS-XRD studies revealed no significant change in the Ni crystallite size, thus preserving Ni dispersion compared with the reduced state. Furthermore, 45 min proved to be sufficient time to obtain relatively good steady-state conversion values. Site time yields (STY: number of molecules of a specific product produced per catalytic site per unit time) followed a similar trend as reactant conversions: STY_H2(KIT‑6/Ni onepot)_= 6.47 s^–1^ > STY_H2(KIT‑6/Ni imp)_= 5.02 s^–1^ ; STY_CO(KIT‑6/Ni onepot)_= 3.28 s^–1^ > STY_CO(KIT‑6/Ni imp)_= 2.63 s^–1^. Moreover, both catalysts showed a relatively low H_2_/CO molar ratio compared to thermodynamically predicted values (Figure S15). This can be ascribed to the accumulation of CO through the RWGS (eq 6) or, as previously mentioned, through the oxidizing surface carbon pathway, also resulting in CO production (inverse eq 3). The presence of the RWGS in our system is consistent with the overall temperature interval of the dry reforming reaction.^44^ Although the RWGS is thermodynamically favored at higher temperatures, it is generally lowered when lower CO_2_ pressures are introduced.^7^ Therefore, under methane-rich conditions, the measured higher CO_2_ conversions (relative to CH_4_) may also arise from the removal of surface carbon. Nevertheless, the molar ratio for KIT-6/Ni onepot recorded after 24 and 50 h tests appeared to be relatively close to that observed for KIT-6/Ni imp after 264 min, which is indicative of a stabilizing effect of Ni-PS in this catalyst.

### 3.4 Post-Mortem Characterization of the KIT-6/Ni onepot

Thermogravimetric analysis was performed for the catalysts used in the synchrotron radiation-assisted experiments (1 h and 45 min catalytic tests for KIT-6/Ni onepot and KIT-6/Ni imp, respectively) and after the 50 h time-on-stream test (Figure 9a). For the latter, two different zones of the catalytic bed were examined, as they varied in color (Figure 9b). For the sake of comparison, thermogravimetric analyses were also carried out for calcined materials. In the measurements, we focused on the 120–900 °C range capturing the most relevant thermal events while excluding mass changes due to physically adsorbed molecules. With the increasing temperature, the mass decreased slowly for the calcined materials, resulting in a total 1.4–2.5% weight loss. This partial weight loss could be also associated with the structural transformations, since the calcined materials were not previously exposed to temperatures above 600 °C. The spent catalysts showed distinct mass losses upon reaching specific temperatures. The spent KIT-6/Ni imp showed a sudden weight loss at ca. 550 °C and subsequently from 550 to 650 °C, which could be related to a partial carbon combustion occurring in two different steps. For this sample, the coke accumulation was significant and in agreement with the observed graphitic-like carbon formation (Figure 7d). The KIT-6/Ni onepot tested for 1 h showed a 6.0% mass loss, while the two bed zones of the catalytic bed after 50 h exhibited a slightly higher mass loss of 11.3% (second zone: 1.4% + third zone: 9.9%). This suggests that carbon formation occurs predominantly during the initial minutes of the catalytic reaction or that some carbon can be removed in situ through parallel reactions. Both scenarios are feasible for our system and will be elaborated further in the following sections. Notably, the clear color distinction across the catalytic bed after the 50 h test suggests an axial evolution of the catalyst’s state in which carbon buildup is mostly observed at the outlet (Figure 9b). Other authors also observed changes across the catalytic bed after DRM. Recently, Giehr et al.^45^ showed that while Ni was reduced along the whole length of the reactor, the oxidation state of cobalt in the model compound CoAl_2_O_4_ was strongly dependent on the axial position in the reactor and the respective spatial partial pressure regime. Similar to our study, different powder colors were recorded across the catalytic bed with the darkest color being at the outlet.

**Figure 9:**
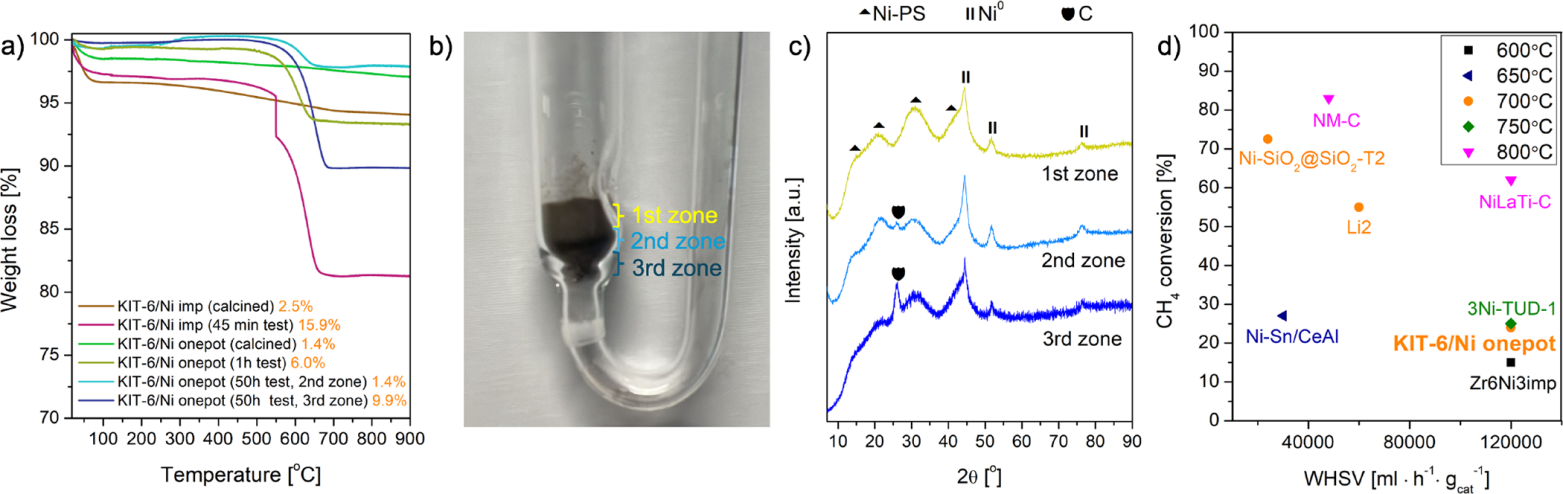
(a) Thermogravimetric analyses carried out for the calcined and spent catalysts. The mass loss for each sample is indicated after the sample name and calculated between 120 and 900 °C. The former temperature corresponds to the steady-state phase, during which the sample was held for 30 min to remove moisture. (b) Catalytic reactor filled with the KIT-6/Ni onepot catalyst after 50 h of methane-rich dry reforming. The image presents the reactor during the cleaning step in which the top and bottom quartz wool plugs were partially removed. The distinct catalyst zones can be observed, each characterized by a different color: 1st zone: gray, 2nd zone: gray-black, and 3rd zone: black. (c) XRD patterns of KIT-6/Ni onepot collected for the spent material after 50 h of testing; diffractograms represent three different zones of the catalytic bed (λ = 0.15406 nm). (d) Performance of KIT-6/Ni onepot compared to previously reported catalysts.

XRD was performed for three different zones of the KIT-6/Ni onepot catalyst after a 50 h test (Figure 9c). The reflection of graphitic carbon (2θ = 26°) was most intense in the third and second zones and absent in the first, indicating a greater presence of coke in the former zones. Moreover, apart from the characteristic reflections of metallic nickel (2θ = 44.5°, 52°, 76.5°), additional broad scattering features were observed at 2θ = 14.2°, 21°, 30.6°, and 41.5°, which are attributed to an amorphous or poorly crystalline nickel phyllosilicate phase. Our data match well the ranges of Ni-PS phases described in the literature, especially considering shifts in the reflection position due to hydration, Ni content, or domain sizes.^25,46,47^ Moreover, a significant increase in the intensity of the Ni-PS signal was observed, suggesting that during the 50 h of DRM, more Ni interacted with silica leading to the formation of a greater amount of nickel phyllosilicate in the catalyst. The distribution of nickel phyllosilicate across the zones appears to be inversely correlated to carbon formation, suggesting that cationic Ni plays a limited role in catalyzing carbon nucleation. However, reliable Rietveld refinement could not be performed due to the extensive overlap of multiple phases in the 2θ range of 10–50°. Consequently, additional analyses are required to confirm this hypothesis. Amorphous silica (SiO_2_) can be also identified by the presence of the broad scattering feature around 2θ of 15–30° that has been masked by more pronounced XRD reflections originating from other phases.

The microscopy study of the KIT-6/Ni onepot catalyst after 24 h of methane-rich dry reforming allowed us to assess the physicochemical changes induced in the reaction conditions (Figure S16). HRTEM observation of the spent catalyst made evident a well-preserved morphology of the nanostructured KIT-6-like support. As shown by in situ XRD, during 1 h of the catalytic reaction, the nickel crystallite size in the Ni phyllosilicate-containing catalysts was stabilized and the presence of crystalline carbon was hardly revealed. After 24 h, multiwalled carbon nanotubes were formed (Figure S16). In general, these graphitic-like carbon nanotubes can arise from methane decomposition (eq 2) and the Boudouard reaction (eq 3) that compete for similar catalytic sites. It is to note that the Boudouard reaction is predominant at *T* < 650 °C, over the reverse reaction.^7^ The sintering of metallic Ni was statistically analyzed in the HRTEM micrographs, indicating that the nickel particles sintered to a minor extent. Small Ni particles anchored in the silica channels showed only a slight increase in the size. The fraction of Ni^0^ crystallites with dimensions below 25 nm constitutes 97.6% of the total nickel crystallite population, while in the spent sample, this fraction decreases to 82.9% (Figure S17). This difference suggests that the nickel embedded within the structural matrix may not undergo significant migration; therefore, it may be an indication of confinement by the KIT-6-like silica support. When examining the 1–25 nm range, only a ca. 15% reduction in the nickel crystallite population is observed, indicating limited sintering, as evidenced by the nearly unchanged mean diameter in such a range. Furthermore, the preservation of cationic species and the particle size of metallic Ni in both reduced and spent samples could contribute to strengthened MSI, which prohibits complete reduction of framework Ni^2+^ sites and, in general, slows down the aggregation of metallic Ni. Similar to the Cu­(II)/Cu­(I) interface in reduced Cu-MFI in the study of Pang et al.,^48^ the unreduced Ni species could be located at the interface between the support and metallic phase, which might stabilize the metallic aggregates.

### 3.5 Enhanced Catalytic Stability Enabled by Thermally Resistant Ni Phyllosilicate with Cationic Ni Species

The findings presented in the preceding text clearly show that the catalytic activity depends directly on the crystallite size and dispersion of Ni but also on other parameters. From the catalyst preparation point of view, one can ask whether special benefits come from the modified KIT-6 synthesis protocol, which would favor the formation of the thermally stable Ni phyllosilicate phase. It is to be noted that there is no such Ni-PS formation behavior previously discussed in the literature. By combining the ammonia evaporation method with a hydrothermal synthesis approach, we successfully produced 1:1 Ni_3_Si_2_O_5_(OH)_4_ for the first time on KIT-6-like silica. The previously reported ammonia evaporation method involved adding ammonia solution in an open system to synthesize nickel phyllosilicates of 2:1 type.^49,50^ KIT-6, SBA-15, and SBA-16 mesoporous silicas use templating approaches with amphiphilic triblock copolymers but differ significantly in the type of structure-directing agents. Mixing n-butanol (KIT-6 protocol) with ethanol (the solvent used for the nickel­(II) hexahydrate precursor) could moderate the polarity and solubility, improving wetting and diffusion of Ni^2+^ into the mesopores.^51^ Furthermore, a more negatively charged surface (due to pH = 9) favored formation of deprotonated silanol groups Si–O^–^, setting up a stage for the layered structure of nickel phyllosilicate. It is to highlight that previously published studies showed that high concentration of surface silanol groups facilitates the formation of nickel phyllosilicates.^24,52^ In our work, isolated surface hydroxyl groups on KIT-6/Ni onepot’s surface could contribute to Ni-PS formation, leading to synthesis of a catalyst with enhanced activity and stability due to the less acidic character.^53^ Indeed, the molecular dynamic simulation of Pfeiffer-Laplaud et al.^54^ performed at the amorphous silica/water interface revealed the pKa of isolated silanols to be 10.3, which is the highest among studied silanol groups. Moreover, a catalyst containing the isolated OH groups seems to have more favorable features responsible for the improved stability in methane dry reforming as investigated in the study of Wang et al. in which a reaction pathway from CH_4_ into syngas by surface OH was suggested (CH_4_ → CH → CHO → CO).^55^ This discussion would be more relevant if the isolated OH groups were not observed for the KIT-6/Ni imp, which is not the case. However, a remarkable difference in the sharpness of the peaks assigned to the isolated silanol groups could have an effect on the catalytic performance. This gives a new insight into already published reports in which it was suggested that advantages of using Ni-PS in DRM are mainly attributed to the superficial OH groups.^26^ Therefore, there must be another parameter responsible for the enhanced catalytic performance of the KIT-6/Ni onepot catalyst. Investigating further, by IR, we reported the different Ni carbonyl groups, revealing the presence of Ni^0^, Ni^+^, and Ni^2+^. It was demonstrated that CO offers much more bonding options with the surface of the KIT-6/Ni onepot than with the counterpart sample. The Ni^+^ and Ni^2+^ centers in the one-pot catalyst are actively involved in CO binding and are not just passive or inert species. One can think of Ni^2+^ being particularly beneficial in the catalytic process and contributing to the reaction mechanism. While Ni^0^ activates methane, properly dispersed and stabilized Ni^2+^ centers on the support can function as Lewis acid sites to activate CO_2_.^56^ Metallic nickel has lower reactivity toward CO_2_ compared to its ionic form, Ni^2+^, which can react rapidly with carbon dioxide to form carbonate species.^57^ Finally, the high pH of 9 led to a drastic change in the textural properties in terms of the specific surface area and modification in the size and shape of mesopores, which could have affected the catalytic behavior. It should be noted that although the 3D pore structure of KIT-6 is widely described as an advantageous feature, it does not appear to strongly determine the catalytic improvement in the dry reforming of methane. Consequently, the significance of possessing a very high *S*_BET_ and well-defined mesopores seems to be low. Besides, the synthesized KIT-6/Ni onepot still remains an amorphous silica structure with the specific surface area comparable to metal oxides commonly used in the methane reforming reactions (e.g., Ni/Al_2_O_3_). Amin et al.^58^ reported that small pore diameters and thin silica walls in the support material were less favorable for Ni catalysts studied in DRM. This leads us to speculate that although it was not possible to estimate the pore wall thickness in KIT-6/Ni onepot from the SAXS results, the material likely possesses a higher average pore wall thickness compared to the counterpart sample. In addition, the formation of larger pores can be considered beneficial, as they can provide easier access for bulkier molecules such as CO_2_ and CH_4_.

Table S4 compares KIT-6/Ni onepot catalysts with several previously reported Ni-based catalysts tested in methane-rich dry reforming. Figure 9d illustrates CH_4_ conversion values reported for differently prepared catalysts tested at various WHSVs and temperatures. CH_4_ conversion was chosen for comparison as CO_2_ tends to participate in the side reactions leading to its extra consumption, e.g., in RWGS. In the discussion, we mainly focus on the comparison between the materials tested at the same WHSV of 120,000 mL/(h ·g_cat_) as our catalyst. Németh et al.^1^ studied Ni/ZrO_2_ catalysts with different loadings of Ni (1 and 3 wt %) and compared them with the 1 wt % Pt/ZrO_2_ catalyst in excess-methane dry reforming (CH_4_:CO_2_:Ar = 68:31:1, 600 °C). During the synthesis, pH was adjusted with NaHCO_3_, which resulted in 0.6 wt % of Na promoter loading in the final catalysts. 3%Ni/ZrO_2_ (Zr6Ni3imp) turned to be the most active catalysts resulting in ca. 15% of CH_4_ conversion and 44% of CO_2_ conversion; however, coke formation was observed on its surface. The isotope labeled measurements showed that 0.142 mg ^12^C and 0.133 mg ^13^C were removed from the sample, which was the highest amount among all of the tested materials. The higher activity of the 3% Ni/ZrO_2_ sample was probably due to the medium size of Ni particles with strong interaction with the support together with the optimal distribution of the Na promoter forwarding part of the surface coke far from the active Ni sites (mostly residing on the support). In this case, the coke did not influence the catalytic conversions and did not poison the sites. 3Ni-TUD-1 (3 wt % of nickel) was studied at 750 °C (CH_4_:CO_2_:N_2_ = 2.1:1:0.3) by Parkhomenko et al.^59^ The authors studied carbon formation by using TPO analysis, stating that after 30 h of testing the amount of carbon was 0.036 mol_coke_ mol^–1^_C_ (mol of deposited carbon per mol of carbon inserted into the reactor, including CO_2_ and CH_4_). The performance of NiLaTi-C was evaluated by Veiga et al.^60^ The authors tested the material at 800 °C in a mixture of CH_4_:CO_2_ = 1.5. The obtained conversions were 62% for CH_4_ and 68% for CO_2_ after 10 h of testing, with ca. 13% of mass loss attributed to the carbon formation. The observed changes in weight loss attributed to carbon formation on our KIT-6/Ni onepot catalyst after 1 h of the synchrotron-assisted reaction and 50 h test were 6.0 and 11.3% (second + third zones), respectively. This may indicate that initially low-ordered carbon (amorphous or defective) nucleated. Moreover, the observed formation of carbon nanotubes after 24 h suggests that carbon continued to grow, while a part of it was simultaneously oxidized, as evidenced by the negligible decrease in CO_2_ and CH_4_ conversions. A comprehensive summary of the carbon analysis is provided in Table S5, which is in line with the previous studies showing that coke deposit can still allow the DRM catalyst to work.^7,61–63^ It should be further mentioned that the conversions values for both CH_4_ and CO_2_ are reasonable compared to the catalytic activity of other catalysts tested at lower WHSVs, i.e., Li2 ^64^ and Ni-SiO_2_@SiO_2_-T2.^65^ Higher WHSVs reduce the residence time of CH_4_ and CO_2_ over the catalyst, leading to lower conversions. The effectiveness of the CO_2_-driven oxidation of surface carbon can also be reduced to some extent, which may contribute to net carbon accumulation.

## 4 Conclusions

For the first time, a thermally stable 1:1 Ni phyllosilicate phase was formed in KIT-6-like silica by employing elevated pH values (pH = 9). In this work, the presence of Ni_3_Si_2_O_5_(OH)_4_ in the reduced samples led to an improved stability in methane-rich dry reforming with cationic nickel species persisting even at high reduction temperatures. In the discussion, attention is given to the role of Ni species (Ni^+^, Ni^2+^) in improving the catalytic activity and stability in methane-rich dry reforming. Balancing catalytic activity with long-term stability, as well as ensuing low carbon formation, is essential. Given the enhanced stability of the KIT-6/Ni onepot catalyst, the deposited graphitic carbon does not lead to deactivation and most likely acts as a reactive intermediate that is partially oxidized during the reaction, indicating effective steady-state control of this catalytic system. Finally, the presence of Ni^+^ and Ni^2+^ shows that the catalytic behavior of Ni-containing KIT-6-like silica also depends on the state of the Ni that can limit coking proceeding via the Boudouard reaction. After 50 h of stable operation in DRM, the catalyst was found to contain an increased quantity of amorphous nickel phyllosilicate, which may be responsible for the improved catalytic stability. Furthermore, identification of the isolated and vicinal silanol bands opens new opportunities for a better understanding of the adsorption mechanism of various molecules, including specific ionic interactions, on the surface of commonly used silica materials.

## Supplementary Material



## Notes

Additional information on catalyst synthesis, TPR-H_2_ procedure, in situ XAS-XRD procedures, catalyst characterization (ICP-OES, N_2_ physisorption, SAXS, TPR-H_2_, STEM-EDS, HRTEM), structural analyses (XRD, XANES, EXAFS), MS data, IR spectra, catalytic performance comparison, and summary of carbon analysis (PDF)

## ∥

Fritz-Haber-Institut der Max-Planck-Gesellschaft, Department of Inorganic Chemistry, Faradayweg 4–6, Berlin 14195, Germany
