# Supplementary material for: In Situ Insights into Ni Phyllosilicate Evolution: Cationic Ni Species as Key to Enhanced Stability in Methane-Rich Dry Reforming
Source: ACS Catal. 2026 Feb 9;16(5):4476–91. doi: 10.1021/acscatal.5c07192 (PMC12973300; doi:10.1021/acscatal.5c07192)
Supplement: Supplementary file 1 [file cs5c07192_si_001.pdf]

Supplementary Information

In Situ Insights into Ni Phyllosilicate Evolution: Cationic Ni Species as Key to Enhanced Stability in Methane-Rich Dry Reforming

Katarzyna Świrk Da Costa,[†*] Paulina Summa,[‡] Marco Fabbiani,[§] Dumitrita Spinu,[†] Valentin Valtchev,[§] Ludovic Pinard,[§] and Magnus Rønning[†]

[†] Norwegian University of Science and Technology, Department of Chemical Engineering, Trondheim 7491, Norway

[‡] Sorbonne Université, CNRS UMR 7190, Institut Jean Le Rond d'Alembert, Saint Cyr l'Ecole 78210, France

[§] Université de Caen, ENSICAEN, CNRS UMR 6506, Laboratoire Catalyse et Spectrochimie, Caen 14000, France

Corresponding Author

* Katarzyna Świrk Da Costa

katarzsw@alumni.ntnu.no

1. Experimental
1.1. Catalysts
1.1.1. One-pot synthesis of Ni phyllosilicates with KIT-6 template

The KIT-6/Ni onepot sample was synthesized at pH of 9 using NH_4OH. The one-pot synthesis strategy is presented in Scheme S1.

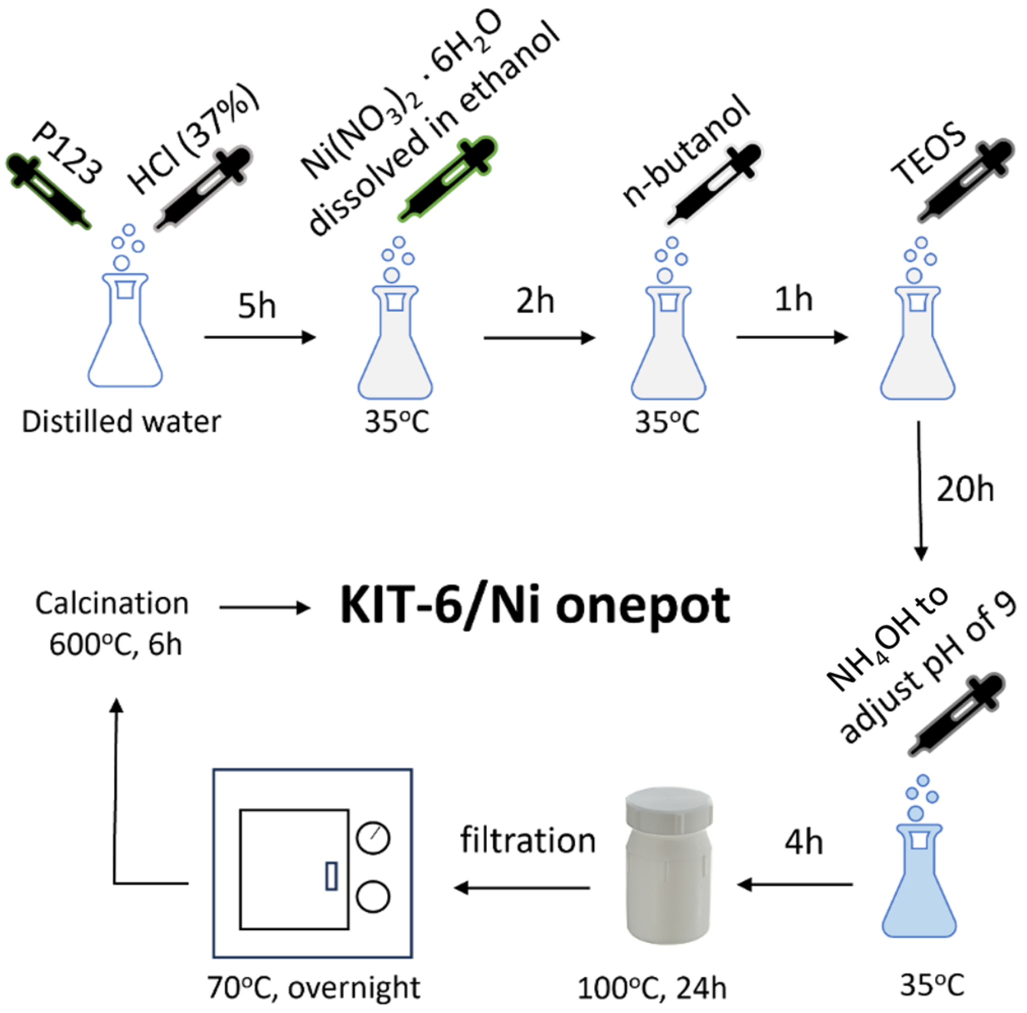

Scheme S1. The one-pot synthesis sequence leading to the formation of mesoporous KIT-6/Ni onepot catalyst, assuming the addition of nickel salt in the first position and adjusting pH to 9 after the solution was aged for 20h at 35°C. P123 refers to Triblock copolymer Pluronic P123 (poly(ethylene glycol)-block-poly(propylene glycol)-block-poly(ethylene glycol). TEOS stands for tetraethyl orthosilicate.

1.2. Characterization methods

The dispersion of Ni estimated by H_2 chemisorption was calculated as:

$$D = 6\frac{\left(\frac{v_m}{a_m}\right)}{d_{va}} \quad \text{(Eq. S1)}$$

Where V_m is the volume occupied by an atom in bulk metal (10.95 Å^3 for Ni), a_m is the area occupied by a surface atom (6.51 Å^2 for Ni) and d_{va} is the mean diameter of metal particle from TEM[1]. During the particle size estimation, 300 particles were counted.

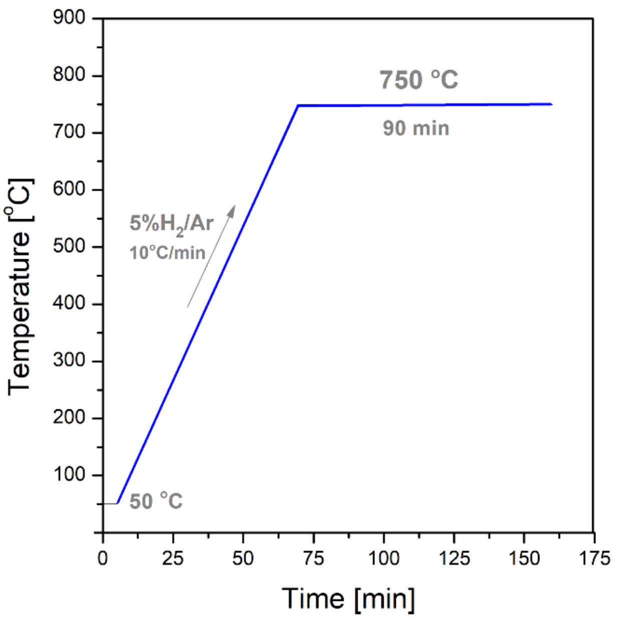

Fig. S1 Procedure applied for the reduction of studied catalysts by using TPR-H_2 technique.

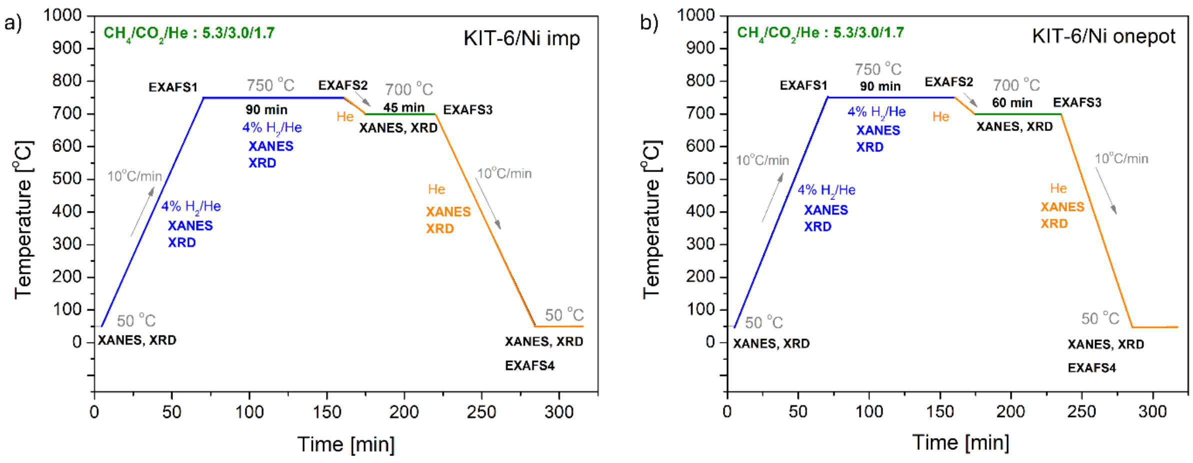

Fig. S2 Experimental procedure followed at the ESRF, Swiss-Norwegian Beamline BM31 including the reduction at 750°C in a mixture of 4%H_2/He for 90 min, cooling step in pure He, and methane-rich dry reforming at 700°C for 45 min (KIT-6/Ni imp) (a), and 60 min (KIT-6/Ni onepot) (b).

2. Results and discussion

2.1. Physiochemical properties of KIT-6/Ni catalysts

The actual contents of Si and Ni in the catalysts were obtained by inductively coupled plasma optical emission spectrometer (ICP-OES) and listed in Table S1. The nickel loading is consistent across the studied catalysts. KIT-6/Ni imp and KIT-6/Ni onepot showed 6.2 and 6.0 wt.% of nickel, respectively. This result allows to make a direct comparison between these two differently prepared catalysts, and confirms that all the assumed nickel amount was introduced during one-pot synthesis.

Table S1. Experimental results of ICP-OES, N_2 physisorption, and small-angle XRD.

Catalyst	Si [wt.%]	Ni [wt.%]	S_{BET} [m²/g][1]	V_{mes} [cm³/g][2]	D_{KJS} [nm][3]	d_{211} [nm][4]	a_0 [nm][5]	L [nm][6]
KIT-6	33.7	n/d	733	0.9	7.8	9.5	23.3	3.85
KIT-6/Ni imp	27.7	6.2	642	0.8	7.6	9.4	24.2	3.90
KIT-6 pH9	33.6	n/d	280	1.2	17.7	-	-	-
KIT-6/Ni onepot	28.9	6.0	240	1.0	19.2	-	-	-

[1] Specific surface area calculated by the BET method
[2] Mesopore volume derived from BJH desorption cumulative volume
[3] Average pore size distribution obtained from BJH desorption corrected with KJS method
[4] (211) reflection plane was taken for calculating d-spacing using the Bragg's law; (211) reflection assigned to n=1
[5] Cubic parameter, $a_0 = d_{211} \cdot \sqrt{h^2 + l^2 + k^2} = d_{211} \cdot \sqrt{6}$
[6] Pore wall thickness, $L = a_0 /2 - D_{KJS}$ [2]

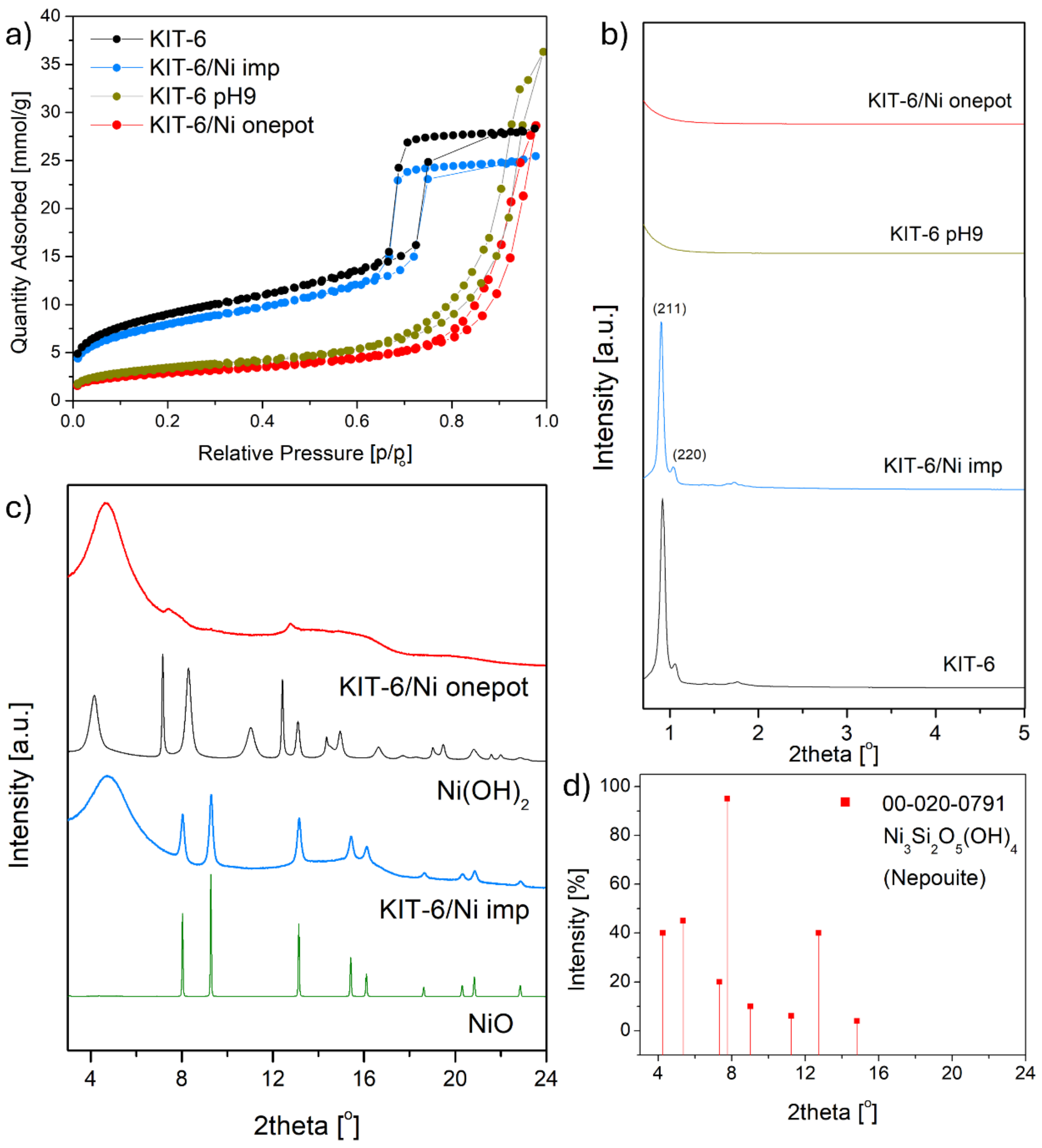

Fig. S3 Isotherms collected through N_2 physisorption (a), small-angle XRD patterns (b), wide-angle XRD patterns (c), and ICDD data of Ni phyllosilicate phase (d).

The N_2 adsorption–desorption isotherms of KIT-6/Ni onepot and KIT-6/Ni imp and their pristine supports (KIT-6 and KIT-6 pH9) are shown in Fig. S3. All isotherms are attributed to typical type IV. For the conventional KIT-6 samples, a H1 hysteresis loop in the relative pressure range of 0.6–1.0 was registered, suggesting the ordered network of interconnected open pores[3]. The samples synthesized at pH=9 displayed H3 subtype hysteresis loops, meaning that slit pores were formed. Moreover, the p/p_0 position of the inflection points were shifted to higher values suggesting relatively broader pore size distribution. Textural properties of the samples are given in Table S1. After the introduction of nickel, a decrease in specific surface area and a decrease in mesopore volume were observed. Such differences between nickel-containing and nickel-free samples can be attributed to the partial blockage of the pores. The pH of the synthesis environment clearly affected the textural properties, leading to lower

surface area, increased volume of mesopores and enlarged pore size. The pronounced decrease in the S_{BET} can also be assigned to the hydrolysis of the KIT-6 mesopore structure, rather than solely being due to the incorporation of Ni[4]. At pH 9, the rate of silica polymerization increases due to the high concentration of hydroxide ions[5]. This tends to produce larger, more uniform pores compared to acidic conditions[6]. Furthermore, the modification with nickel led to much lower specific surface area and reduced volume of mesopores resulting from the partial blockage caused by nickel species. An expansion of pore size was observed, possibly from isomorphic substitution of nickel atoms into silica frameworks, which leads to significant shrinkage of the walls and, consequently, to pore expansion[4].

The small-angle XRD patterns indicated importance of the pH values on the structure of the synthesized materials (Fig. S3b). KIT-6/Ni imp resolved the diffraction peaks at 2θ angles below 2°, corresponding to (211) and (220) planes, which is in good agreement with the XRD pattern of the parent KIT-6 demonstrating a typical structural order with the bicontinuous cubic $Ia3\overline{d}$ space group [6–8]. The peak position does not shift considerably after impregnation with nickel. Moreover, the width of the d_{211} peak is similar for both samples, suggesting a comparable degree of structural ordering after the impregnation[9]. In contrast, KIT-6 pH9 and KIT-6/Ni onepot did not show any diffraction peaks in the range below 2°. The alkaline pH caused the degradation of the mesoporous silica's structural regularity and/or $Ia3\overline{d}$ structure to some extent. Wall thickness and related parameters were calculated for the catalysts revealing the diffractions peaks (Table S1). The estimated values are 3.85 and 3.90 nm for KIT-6 and KIT-6/Ni imp, respectively. Kishor and Ghoshal[9] found wall thickness of 3.55 nm for the KIT-6 synthesized at 60°C (S_{BET} = 733 m^2/g, pore diameter 4.9 nm, pore volume 0.77 cm^3/g) and 3.54 nm for the one synthesized at 100°C (S_{BET} = 857 m^2/g, pore diameter 6.6 nm, pore volume 1.25 cm^3/g). Wall thickness is a critical parameter influencing other textural properties, which include specific surface area, pore size and pore volume. The unit-cell parameters (a_0) varied from 20.57 - 24.0. Furthermore, in the work of Aslam et al.[10], d_{211} and a_0 for KIT-6 were observed to be similar to those reported in our study, i.e., 9.4 nm and 23 nm, respectively. The authors did not estimate the pore thickness of the walls. The effect of Ni addition assuming three different nickel precursors (NO_3^-, Cl^-, or Ac^-) with the same metal loading in each sample (30 wt.%) was investigated. The sample impregnated with $Ni(NO_3)_2{\cdot}6H_2O$ (IWI), named as Ni/KIT-6(NO_3^-), did not show significant differences compared to the bare support, manifesting d_{211}=9.41 nm and a_0=23 nm. This shows that our results are largely consistent with observations made in previously published studies.

The wide-angle XRD patterns were collected for the calcined samples to identify the existing phases in the studied samples (Fig. S3c). Both KIT-6/Ni imp and KIT-6/Ni onepot generated a broad peak at 2θ of 4.8°, attributed to amorphous silica. The recorded diffractograms were compared with the measured standards, i.e., NiO and $Ni(OH)_2$. While a good match with NiO can be found for KIT-6/Ni imp, the formation of a different crystalline phase was observed for KIT-6/Ni onepot. Interestingly, typical diffraction peaks for crystalline nickel phyllosilicate $Ni_3Si_2O_5(OH)_4$, posing a structure of nepouite (1:1) with *Pnma* space group, were featured (Fig. S3d). The use of increased pH environment facilitated formation of the latter phase. Probably during calcination, a certain fraction of Ni^{2+} migrated from the KIT-6 mesopores to the outer surface and formed nickel phyllosilicate fibrous structure (further confirmed by microscopy studies). An exsolution of the $Ni_3Si_2O_5(OH)_4$ phase after calcination was observed in previously published reports, i.e., Ni/SiO_2[11], Ni-7Mg/SBA-15-AE[12], or Ni/SBA-16-AE[13]. On the other hand, depending on the synthesis protocol, for specific catalysts $Ni_3Si_2O_5(OH)_4$ could only be detected in the uncalcined materials consisting the layered Ni-PS structure, which tended to collapse after a thermal treatment and resulted in the presence of SiO_2 and NiO[14,15]. This behavior is similar to that of hydrotalcites, being able to decompose

and form mixed oxides[16]. In the current study, the XRD pattern of the uncalcined material did not show presence of crystalline $Ni_3Si_2O_5(OH)_4$ (Fig. S4).

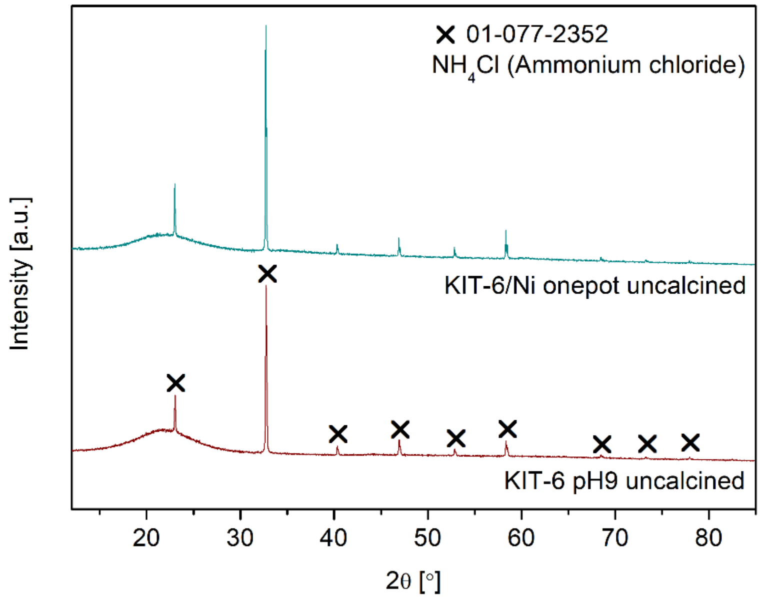

Fig. S4 XRD pattern of uncalcined KIT-6/Ni onepot.

The tested materials showed a presence of amorphous silica (2θ of ca. 23°) and a crystalline phase of NH_4Cl (ICDD 01-077-2352) which diffraction peaks are marked in Fig. S4. Ammonium chloride can be used as an alkaline reagent for the ammonia evaporation to synthesize nickel phyllosilicates[17]. In our case, added ammonia reacted with already present HCl to form crystalline NH_4Cl. This compound decomposes in air at around 345°C into ammonia and hydrochloric acid ($NH_3Cl \rightarrow NH_3 + HCl$)[18].

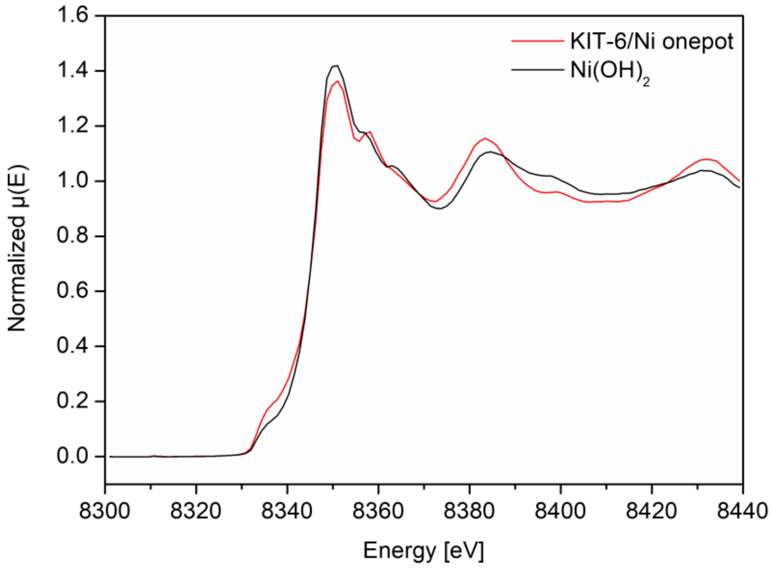

Fig. S5 XANES of KIT-6/Ni onepot and $Ni(OH)_2$ standard.

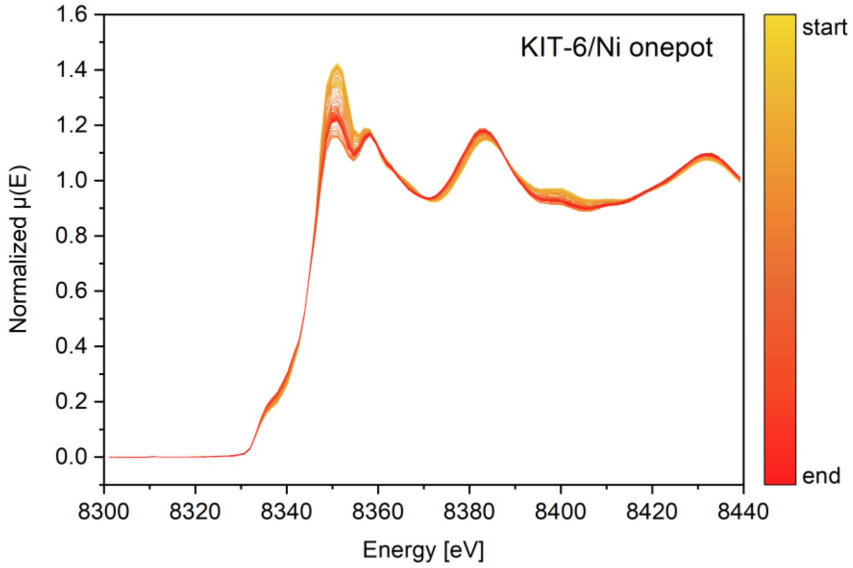

Fig. S6 Overview of XANES changes collected *in situ* during reduction of KIT-6/Ni onepot, i.e., ramping up from 50 to 750°C and keeping the sample for 90 min at 750°C during reduction in a mixture of 4% H_2/He.

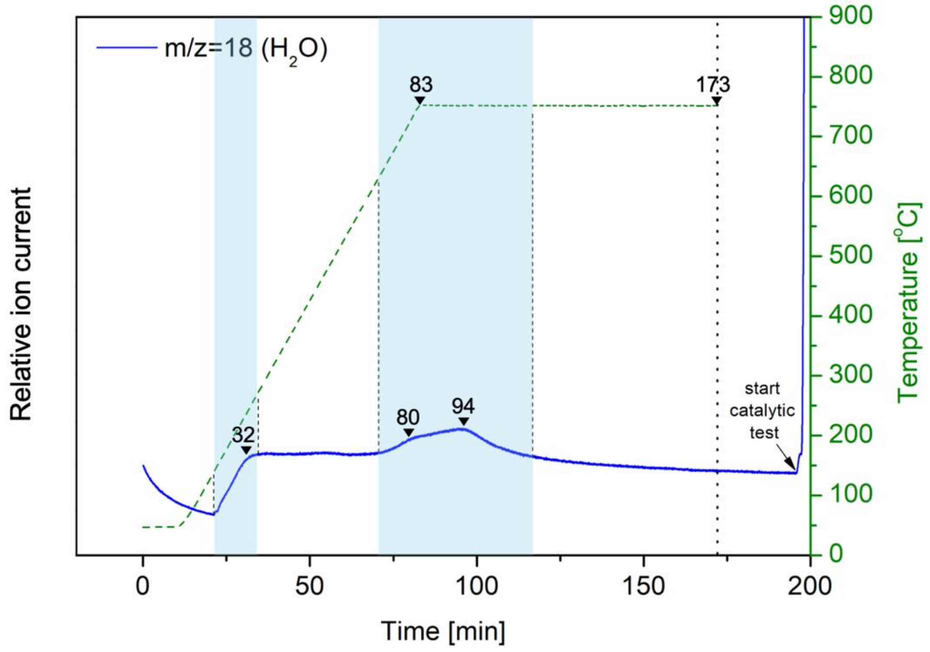

Fig. S7 Changes of m/z=18 (H_2O) signal in a function of time and temperature during the reduction course (KIT-6/Ni onepot).

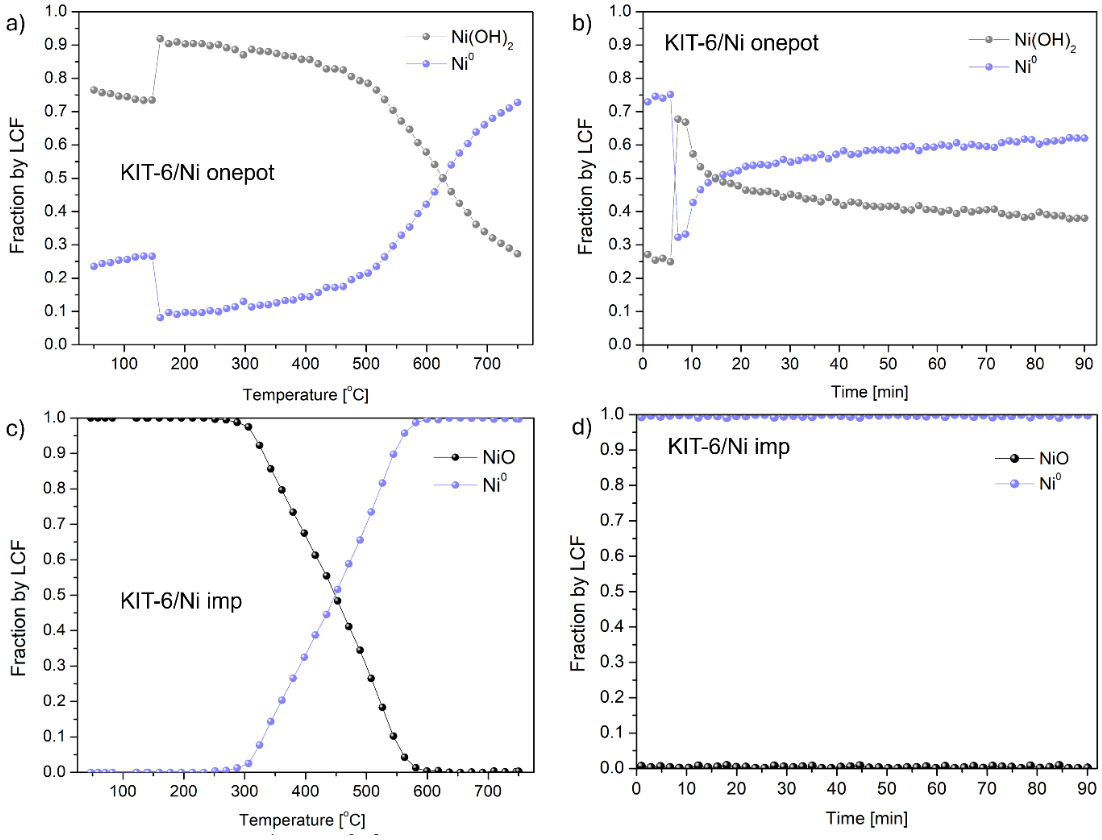

Fig. S8 Linear combination fitting (LCF) of XANES collected for KIT-6/onepot (a,b) and KIT-6/Ni imp (c,d). The Y axis represents weight fraction.

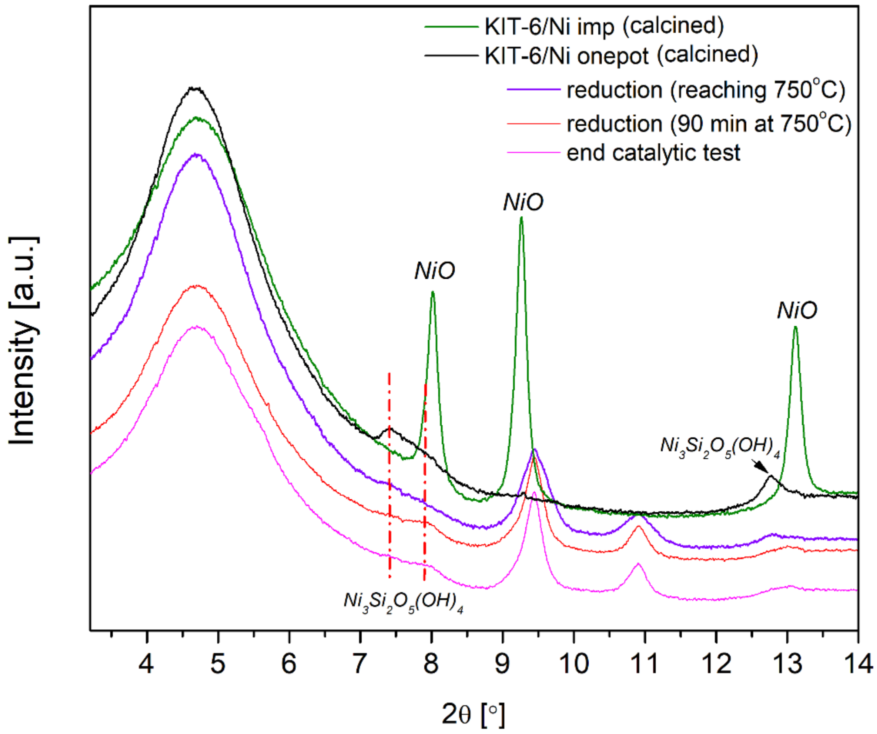

Fig. S9 Comparison of X-ray diffractograms to determine presence of Ni phyllosilicate phase.

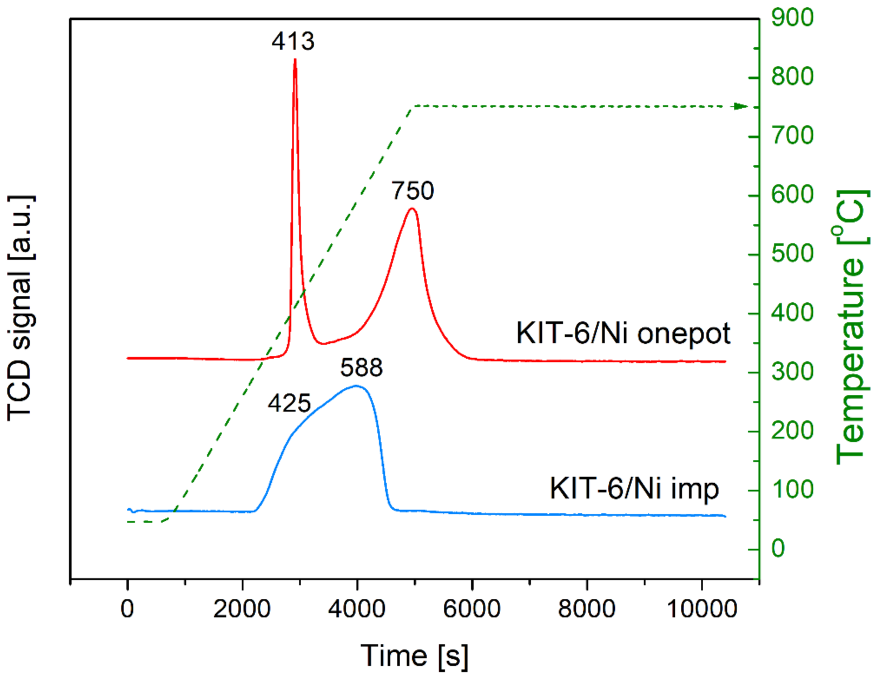

Fig. S10 TPR-H_2 profiles of calcined KIT-6/Ni onepot and KIT-6/Ni imp.

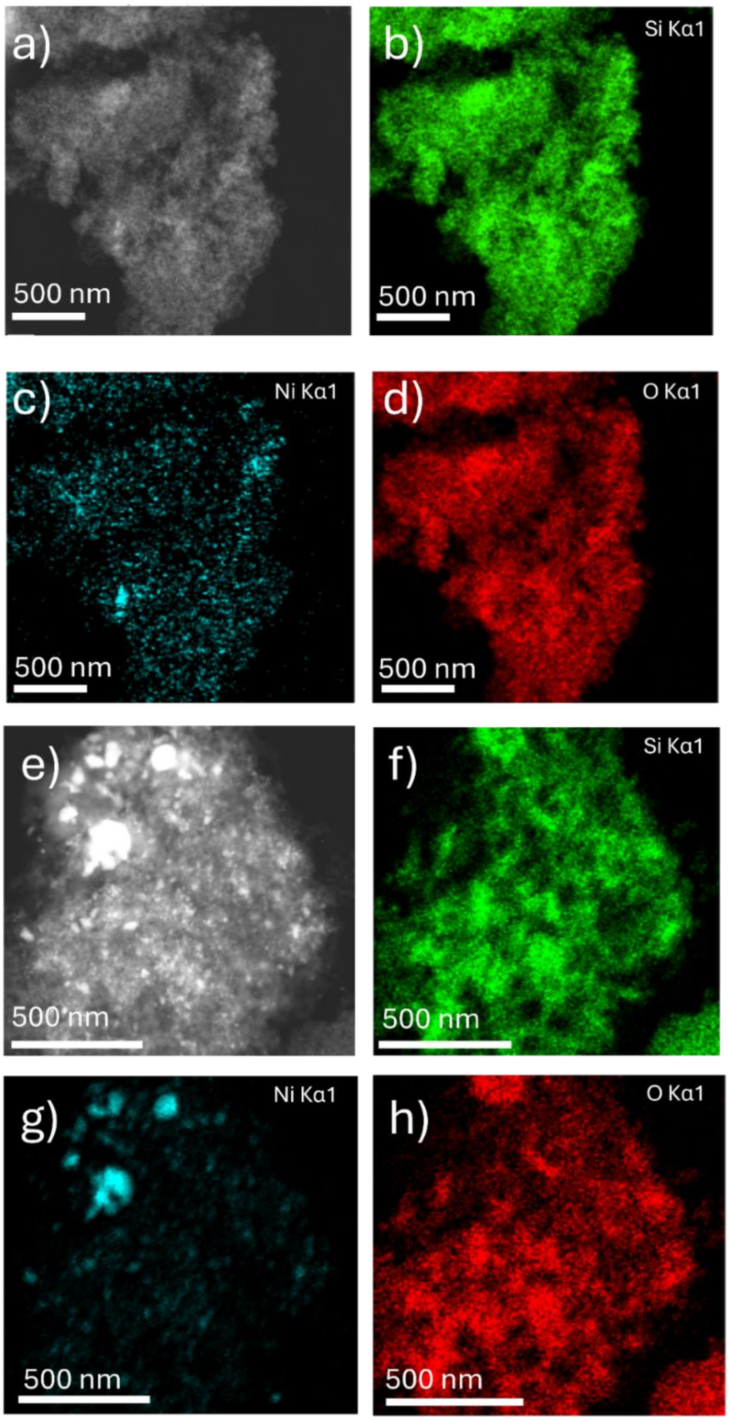

Fig. S11 STEM-EDS micrographs of the KIT-6/Ni onepot catalyst reduced at 750°C for 90 min in a mixture of 5%H_2/Ar. Elemental maps include distribution of Si, Ni, O.

STEM-EDS images of the pre-reduced catalysts showed well dispersed Ni metal particles which are marked as white circular contrast (Fig. S11). The EDS elemental mapping displayed even distribution of silicon, nickel, and oxygen elements in the modified samples. Larger particles of metallic nickel were observed (Fig. S11e,g), probably originating from Ni-PS formed on the KIT-6 surface being able to reduce at relatively low temperature of ca. 400°C.

Table S2 Structural parameters from the Ni K-edge EXAFS analysis for KIT-6/Ni onepot and KIT-6/Ni imp catalysts after reduction in a mixture of 4%H_2/He at 750°C for 90 min.

Catalyst	State	Bond	CN	R [Å]	$\sigma^2 \cdot 100$ [$Å^2$]	R-factor
KIT-6/Ni onepot	Reduced	Ni − Ni	12.0±0.1	2.5±0.01	0.8±0.1	0.010
KIT-6/Ni imp	Reduced	Ni − Ni	12.0±0.1	2.5±0.01	0.8±0.1	0.023

The k^3-weighted Fourier transform EXAFS spectra of KIT-6/Ni onepot and KIT-6/Ni imp are presented in R-space in Fig. S12. The spectra resembled those of the Ni foil implying that H_2 reduction resulted in the formation of predominantly metallic Ni. The presence of the peak in the first coordination shell can certainly be attributed to the Ni − Ni bond (at around 2.5Å). The Ni−Ni peak became as intense as the analogous peak for the Ni foil with a coordination number near 12 (Fig. S12). The fitting results including coordination numbers (CN), interatomic distances (R), Debye-Waller factors (σ^2), R-factors are listed in Table S2. Although in the KIT-6/Ni onepot catalyst a certain fraction of Ni species remained within the Ni phyllosilicate, and retained their initial cationic state, this was not revealed by EXAFS. This is most likely due to a minor contribution of Ni^{2+} appearing to be below the detection limit of this characterization technique. According to the literature, EXAFS of Ni phyllosilicates can reveal the presence of Ni − Si bond in the first shell[12].

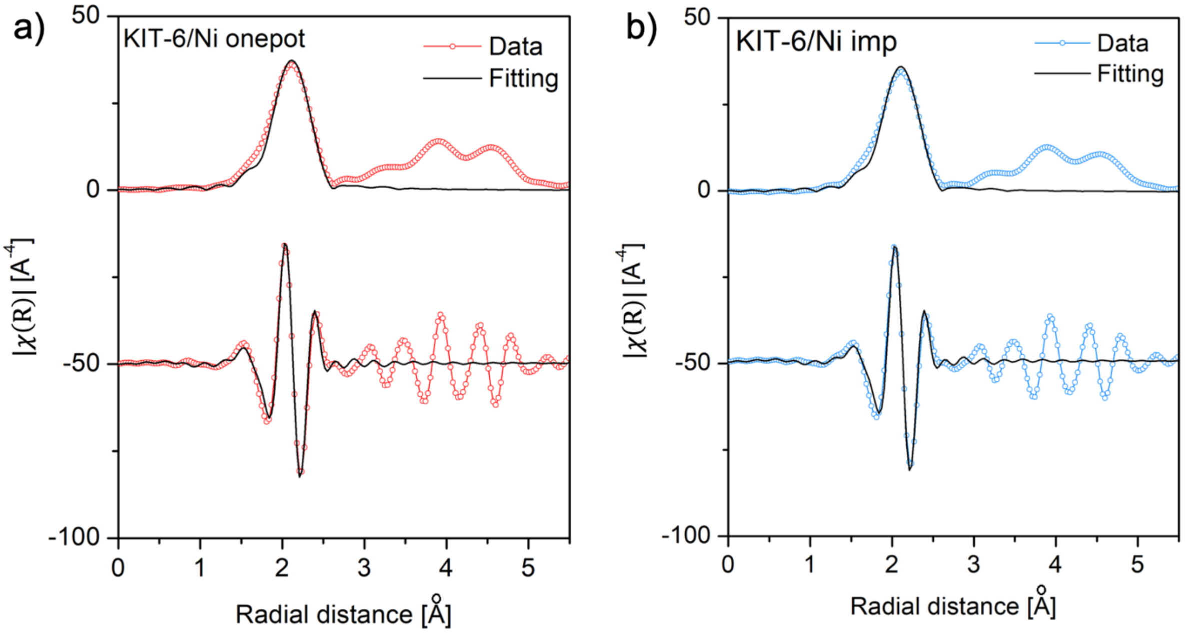

Fig. S12 Ni K-edge Fourier-transformed k^3-weighted EXAFS of the reduced catalysts (in a mixture of 4%H_2/He at 750°C for 90 min) presented in magnitude and real. Spectra are vertically offset to allow for better visualization. The data is not corrected for phase shifts.

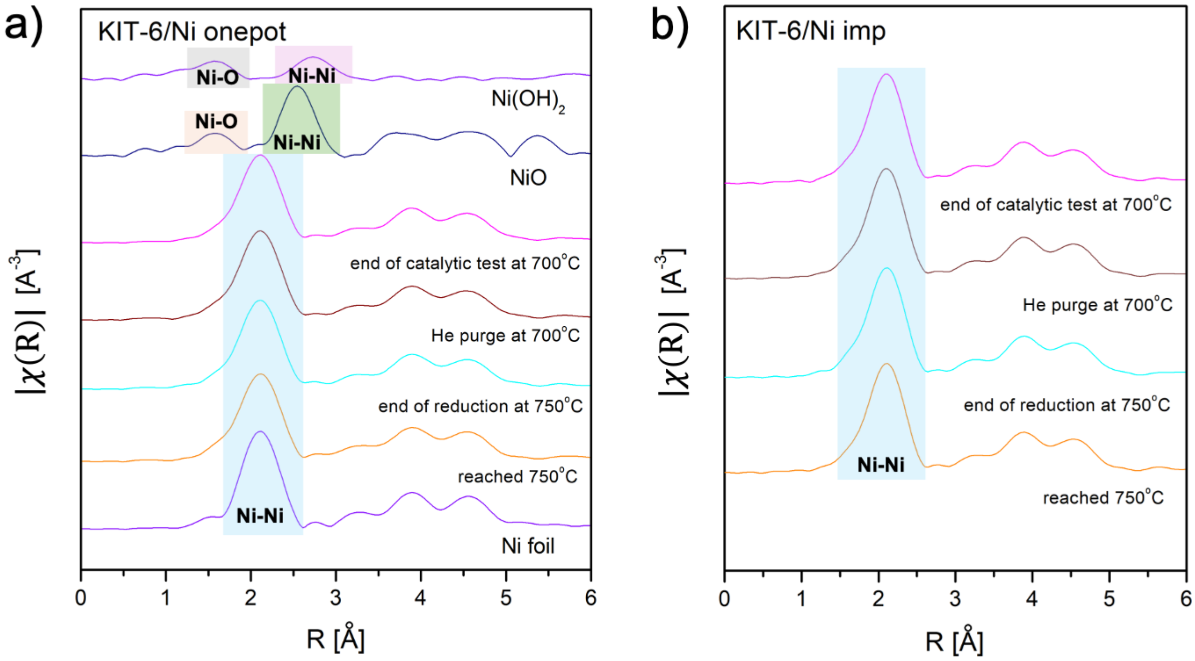

Fig. S13 Ni K-edge Fourier-transformed k^3-weighted EXAFS of the different steps of catalyst's investigation during *in situ* XAS-XRD. Spectra are vertically offset to allow for better visualization. The data is not corrected for phase shifts.

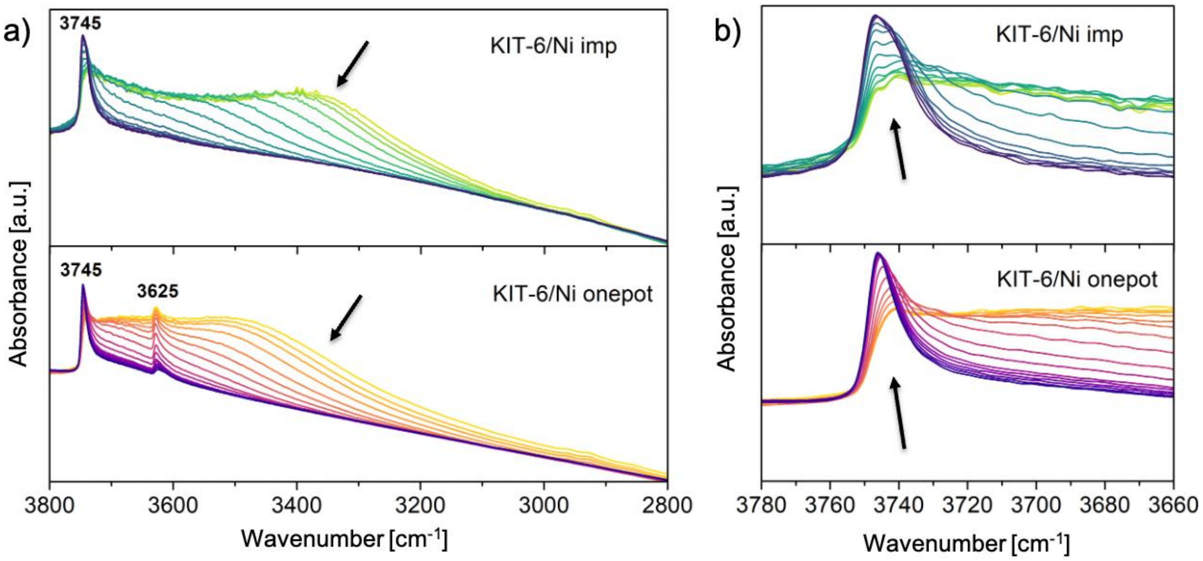

Fig. S14 IR spectra of pretreatment carried out from RT to 650°C (a) full collected range (b) magnification.

Table S3 Estimated experimental error for KIT-6/Ni onepot catalyst based on the standard deviation of the number of conducted tests.

Duration of test	XCH_4 [%]	Exp. error CH_4 (± SD) [%]	XCO_2 [%]	Exp. error CO_2 (± SD) [%]
45 min	30.2	± 2.8	54.1	± 2.8
45 min	28.6		52.8	
45 min	34.1		58.2	
1h	33.9	± 2.8	57.7	± 2.6
1h	28.5		52.6	
1h	29.9		54.2	
24h	24.7	± 0.8	47.3	± 1.3
24h	25.8		49.2	
50h	24.4	GC calibration ca. ±1%	47.1	GC calibration ca. ±1%

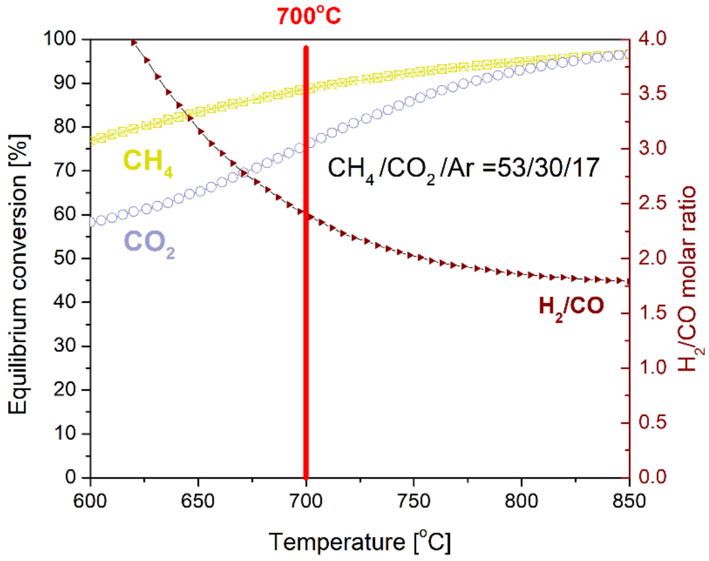

Fig. S15 Thermodynamic equilibrium conversion values for $CH_4/CO_2/Ar$ = 5.3/3.0/1.7 performed in HSC Chemistry software.

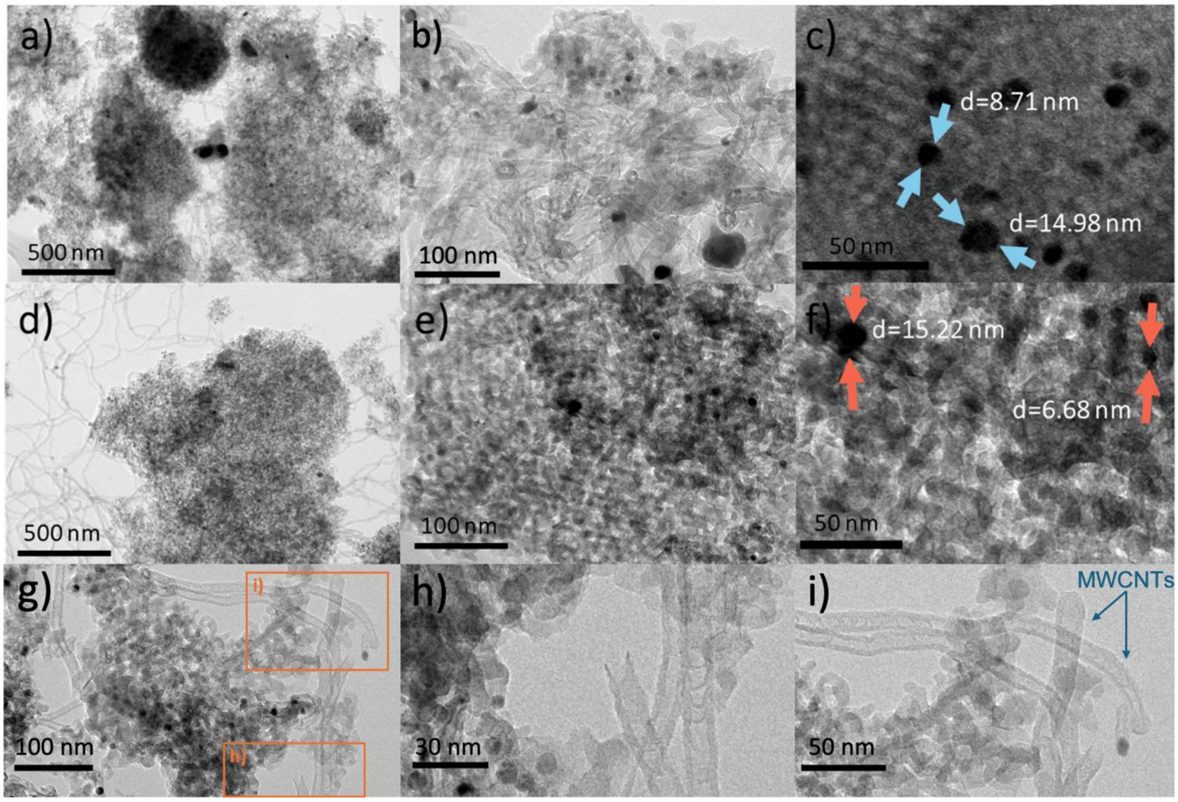

Fig. S16 HRTEM of KIT-6/Ni onepot used in the catalytic test for 24 h.

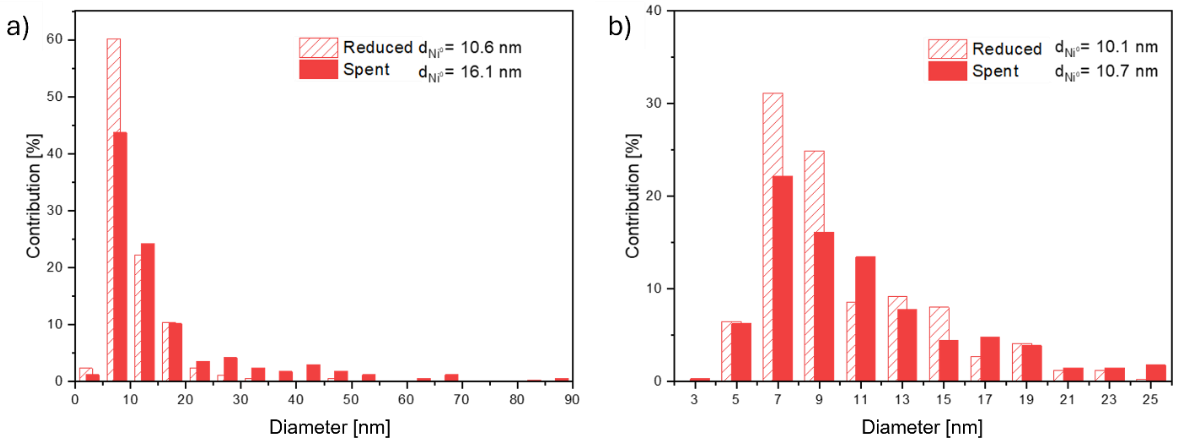

Fig. S17 Histograms of nickel particles counted for the reduced and spent KIT-6/Ni onepot.

Table S4. Catalytic performance of various Ni-containing catalysts tested in methane-rich dry reforming at atmospheric pressure.

No	Catalyst	Ni [wt.%]	T [°C], time[h]	Gas mixture	WHSV [$ml\cdot h^{-1}\cdot g_{cat}^{-1}$]	CH_4 conv. [%]	CO_2 conv.[%]	Carbon from TGA	Ref.
1	KIT-6/Ni onepot	6 wt.%	700°C, 45 min, 24h	CH_4:CO_2:Ar = 53:30:17<br>CH_4/CO_2=1.8	120,000	30.8±2.8% (1h)<br>24.4±1% (50h)	54.8±2.6% (1h)<br>47.1±1% (50h)	6.0 % (1h)<br>11.3 % (2nd plus 3rd zone)	This work
2	Zr6Ni3 imp<br>(3wt.% Ni/ZrO_2)	3 wt.%	600°C, 2h	CH_4:CO_2:Ar = 68:31:1<br>CH_4/CO_2=2.19	120,000	~15%	~44%	No TGA data; TPO showed that carbon can be removed at 490°C. The isotope labeled measurements showed that 0.142 mg ^{12}C, and 0.133 mg ^{13}C was removed during TPO	[19]
3	NiLaTi-C	11.9 wt.% (NiO)	800°C, 10h	CH_4/CO_2=1.5	120,000	~62%	~68%	ca. 13% mass loss, 87% remaining after 10h	[20]
4	Li2	77.4 wt.%	700°C, 480 min = 8h	1.5:1:7.5 for CH_4:CO_2:N_2	60,000	55%	~92%	ca. 93% mass loss, 7% remaining after 480 min	[21]
5	NM-C	ca. 30 wt.%	800°C, 24h	60% CH_4 40% CO_2, 400 ml/min CH_4/CO_2=1.5	48,000	~83%	~95%	91.1% mass loss, however 85% was mass loss registered for the used Haydale® CNT support	[22]
6	Ni-SiO_2@ SiO_2-T2	10 wt.%	700°C, 20h	CH_4/CO_2 = 1.5	24,000	72.5%	93%	No TGA data, according to FESEM analysis the carbon content is negligible	[23]
7	3Ni-TUD-1	3 wt.%	750°C, 30h	CH_4:CO_2:N_2 = 2.1:1:0.3	120,000	25 %	45 %	TPO showed low as 0.036 mol_{coke} mol_{C}^{-1} carbon formation	[24]
8	Ni-Sn/CeAl	10.3 wt.% (NiO)	650°C, 20h	CH_4/CO_2=1.5	30,000	27%	~62%	26.81% of mass loss	[25]

Table S5. Carbon formed on the KIT-6/Ni onepot catalyst.

Test type	Duration	Technique for C analysis	Observation	Feasible conclusion
In situ experiments at synchrotron	1h	XRD (Fig. 7c)	No XRD (002) peak at 2θ of ca. 5.8°	Hardly detected presence of C peak, possibly due to poor graphitization, e.g., the presence of amorphous or defective carbon. The carbon content may be below the detection limit of XRD. The beam was positioned in the middle of the catalytic bed
		TGA (Fig. 9a)	6% weight loss, oxidation onset at ca. 550°C	The onset temperature suggests the presence of moderately disordered carbon. TGA recorded its presence. It should be noted that the TGA analysis was performed using the entire catalytic bed, including regions near the reactor outlet where higher carbon deposition may have occurred
Catalytic tests in lab	First hour - analyzed within 264 min test	microGC (Fig. 8a)	First 30 min: a decrease in conversion,<br>From 30 min – 60 min: the conversion values become stabilized	The observed decrease in conversion may be attributed to the initial deposition of disordered carbon prior to reaching steady-state operation. Under steady-state conditions, carbon may occur, however, it is most likely simultaneously oxidized *in situ*
Catalytic tests in lab	24h	microGC (Fig. 8d)	Relatively steady conversion values	Carbon formation and its simultaneous oxidation via side reactions, therefore the measured conversion does not decrease significantly
		HRTEM (Fig. S16)	MWCNTs	Carbon nanotubes developed over 24h, indicating that the carbon population accumulate over time compared to the state after 1h test
Catalytic tests in lab – different colors were observed across the catalytic bed after this test (Fig. 9b)	50h	microGC (Fig. 8e)	Relatively steady conversion values	A plausible explanation is that, during the steady-state, carbon is continuously formed with part of it simultaneously oxidized *in situ*. Consequently, the measured conversion does not decrease significantly
		TGA (Fig. 9a)	2nd zone: 1.4%<br>3rd zone: 9.9%<br>Sum: 11.3%	Net carbon accumulation (formation > oxidation over time) indicates continued carbon deposition
		XRD (Fig. 9c)	1st zone: no graphitic C peak detected,<br>2nd zone: C peak present,<br>3rd zone: C peak is more intense than in the 2nd zone	XRD analysis indicated variations in the distribution of carbon across the catalytic bed with the lowest carbon formation present at the reactor inlet

References

(1) Bergeret, G.; Gallezot, P. Handbook of Heterogeneous Catalysis; Ertl, G., Knozinger, H., Weitkamc, J., Eds.; Wiley-VCH, 1997; pp 439–453.

(2) Wang, W.; Qi, R.; Shan, W.; Wang, X.; Jia, Q.; Zhao, J.; Zhang, C.; Ru, H. Synthesis of KIT-6 Type Mesoporous Silicas with Tunable Pore Sizes, Wall Thickness and Particle Sizes via the Partitioned Cooperative Self-Assembly Process. *Microporous Mesoporous Mater.* **2014**, *194*, 167–173. https://doi.org/10.1016/j.micromeso.2013.10.028.

(3) Schlumberger, C.; Thommes, M. Characterization of Hierarchically Ordered Porous Materials by Physisorption and Mercury Porosimetry—A Tutorial Review. *Adv. Mater. Interfaces* **2021**, *8* (4). https://doi.org/10.1002/admi.202002181.

(4) Liu, Z.; Zhou, J.; Cao, K.; Yang, W.; Gao, H.; Wang, Y.; Li, H. Highly Dispersed Nickel Loaded on Mesoporous Silica: One-Spot Synthesis Strategy and High Performance as Catalysts for Methane Reforming with Carbon Dioxide. *Appl. Catal. B Environ.* **2012**, *125*, 324–330. https://doi.org/10.1016/j.apcatb.2012.06.003.

(5) Goto, K. Effect of PH on Polymerization of Silicic Acid. *J. Phys. Chem.* **1956**, *60* (7), 1007–1008.

(6) Li, B.; Luo, X.; Huang, J.; Wang, X.; Liang, Z. One-Pot Synthesis of Ordered Mesoporous Cu-KIT-6 and Its Improved Catalytic Behavior for the Epoxidation of Styrene: Effects of the pH Value of the Initial Gel. *Cuihua Xuebao/Chinese J. Catal.* **2017**, *38* (3), 518–528. https://doi.org/10.1016/S1872-2067(17)62767-0.

(7) Dinari, M.; Mohammadnezhad, G.; Nabiyan, A. Preparation and Characterization of Nanocomposite Materials Based on Polyamide-6 and Modified Ordered Mesoporous Silica KIT-6. *J. Appl. Polym. Sci.* **2016**, *133* (10), 1–6. https://doi.org/10.1002/app.43098.

(8) Cao, H. X.; Zhang, J.; Guo, C. L.; Chen, J. G.; Ren, X. K. Modifying Surface Properties of KIT-6 Zeolite with Ni and V for Enhancing Catalytic CO Methanation. *Appl. Surf. Sci.* **2017**, *426*, 40–49. https://doi.org/10.1016/j.apsusc.2017.07.138.

(9) Kishor, R.; Ghoshal, A. K. Understanding the Hydrothermal, Thermal, Mechanical and Hydrolytic Stability of Mesoporous KIT-6: A Comprehensive Study. *Microporous Mesoporous Mater.* **2017**, *242*, 127–135. https://doi.org/10.1016/j.micromeso.2017.01.020.

(10) Aslam, S.; Subhan, F.; Yan, Z.; Peng, P.; Qiao, K.; Xing, W.; Bai, P.; Ullah, R.; Etim, U. J.; Zeng, J.; Ikram, M. Facile Fabrication of Ni-Based KIT-6 for Adsorptive Desulfurization. *Chem. Eng. J.* **2016**, *302*, 239–248. https://doi.org/10.1016/j.cej.2016.05.041.

(11) Zhang, Y.; Liu, Q. Nickel Phyllosilicate Derived Ni/SiO_2 catalysts for CO_2 methanation: Identifying Effect of Silanol Group Concentration. *J. CO2 Util.* **2021**, *50*, 101587. https://doi.org/10.1016/j.jcou.2021.101587.

(12) Hongmanorom, P.; Ashok, J.; Zhang, G.; Bian, Z.; Wai, M. H.; Zeng, Y.; Xi, S.; Borgna, A.; Kawi, S. Enhanced Performance and Selectivity of CO_2 Methanation over Phyllosilicate Structure Derived Ni-Mg/SBA-15 Catalysts. *Appl. Catal. B Environ.* **2021**, *282*, 119564. https://doi.org/10.1016/j.apcatb.2020.119564.

(13) Sun, C.; Summa, P.; Wang, Y.; Świrk Da Costa, K.; Miró i Rovira, A.; Casale, S.; Świerczek, K.; Hu, C.; Rønning, M.; Da Costa, P. Boosting CO_2 Reforming of Methane via the Metal-Support Interaction in Mesostructured SBA-16-Derived Ni Nanoparticles. *Appl. Mater. Today* **2022**, *26*. https://doi.org/10.1016/j.apmt.2021.101354.

(14) Sivaiah, M. V.; Petit, S.; Barrault, J.; Batiot-Dupeyrat, C.; Valange, S. CO_2 Reforming of CH_4 over Ni-Containing Phyllosilicates as Catalyst Precursors. *Catal. Today* **2010**, *157* (1–4), 397–403. https://doi.org/10.1016/j.cattod.2010.04.042.

(15) Sivaiah, M. V.; Petit, S.; Beaufort, M. F.; Eyidi, D.; Barrault, J.; Batiot-Dupeyrat, C.; Valange, S. Nickel Based Catalysts Derived from Hydrothermally Synthesized 1:1 and 2:1 Phyllosilicates as Precursors for Carbon Dioxide Reforming of Methane. *Microporous Mesoporous Mater.* **2011**, *140* (1–3), 69–80. https://doi.org/10.1016/j.micromeso.2010.09.015.

(16) Świrk, K.; Rønning, M.; Motak, M.; Grzybek, T.; Da Costa, P. Synthesis Strategies of Zr- and Y-Promoted Mixed Oxides Derived from Double-Layered Hydroxides for Syngas Production via Dry Reforming of Methane. *Int. J. Hydrogen Energy* **2020**, https://doi.org/10.1016/j.ijhydene.2020.04.239.

(17) Ma, B.; Cui, H.; Wang, D.; Wu, P.; Zhao, C. Controllable Hydrothermal Synthesis of Ni/H-BEA with a Hierarchical Core-Shell Structure and Highly Enhanced Biomass Hydrodeoxygenation Performance. *Nanoscale* **2017**, *9* (18), 5986–5995. https://doi.org/10.1039/c7nr00471k.

(18) Zelenková, G.; Slovák, V. Decomposition of Ammonium Salts by Quantitative TG-MS. *J. Therm. Anal. Calorim.* **2022**, *147* (24), 15059–15068. https://doi.org/10.1007/s10973-022-11747-0.

(19) Németh, M.; Schay, Z.; Srankó, D.; Károlyi, J.; Sáfrán, G.; Sajó, I.; Horváth, A. Impregnated Ni/ZrO_2 and Pt/ZrO_2 Catalysts in Dry Reforming of Methane: Activity Tests in Excess Methane and Mechanistic

Studies with Labeled 13CO_2. *Appl. Catal. A Gen.* **2015**, *504*, 608–620. https://doi.org/10.1016/j.apcata.2015.04.006.
(20) Veiga, S.; Romero, M.; Faccio, R.; Segobia, D.; Apesteguía, C.; Laura Pérez, A.; Dante Brondino, C.; Bussi, J. Biogas Dry Reforming over Ni-La-Ti Catalysts for Synthesis Gas Production: Effects of Preparation Method and Biogas Composition. *Fuel* **2023**, *346*. https://doi.org/10.1016/j.fuel.2023.128300.
(21) Steffens, C. M.; Perez-Lopez, O. W. Biogas Dry Reforming over Li–Ni–Al LDH-Derived Catalysts. *Int. J. Hydrogen Energy* **2024**, *71*, 205–216. https://doi.org/10.1016/j.ijhydene.2024.05.285.
(22) Saconsint, S.; Srifa, A.; Koo-Amornpattana, W.; Assabumrungrat, S.; Sano, N.; Fukuhara, C.; Ratchahat, S. Development of Ni–Mo Carbide Catalyst for Production of Syngas and CNTs by Dry Reforming of Biogas. *Sci. Rep.* **2023**, *13* (1), 1–18. https://doi.org/10.1038/s41598-023-38436-8.
(23) Kaviani, M.; Rezaei, M.; Alavi, S. M.; Akbari, E. Biogas Dry Reforming over Nickel-Silica Sandwiched Core–Shell Catalysts with Various Shell Thicknesses. *Fuel* **2024**, *355*, 129533. https://doi.org/10.1016/j.fuel.2023.129533.
(24) Parkhomenko, K.; Tyunyaev, A.; Martinez Tejada, L. M.; Komissarenko, D.; Dedov, A.; Loktev, A.; Moiseev, I.; Roger, A. C. Mesoporous Amorphous Silicate Catalysts for Biogas Reforming. *Catal. Today* **2012**, *189* (1), 129–135. https://doi.org/10.1016/j.cattod.2012.03.057.
(25) Le Saché, E.; Johnson, S.; Pastor-Pérez, L.; Horri, B. A.; Reina, T. R. Biogas Upgrading via Dry Reforming over a Ni-Sn/CeO_2-Al_2O_3 Catalyst: Influence of the Biogas Source. *Energies* **2019**, *12* (6). https://doi.org/10.3390/en12061007.
